# Snail promotes the generation of vascular endothelium by breast cancer cells

**DOI:** 10.1038/s41419-020-2651-5

**Published:** 2020-06-15

**Authors:** Zhenyu Chang, Yanan Zhang, Jie Liu, Yiqiong Zheng, Huayue Li, Yanjun Kong, Pengyun Li, Haiwen Peng, Yajiao Shi, Bo Cao, Fang Ran, Yingjie Chen, Yuhua Song, Qinong Ye, Lihua Ding

**Affiliations:** 1 Department of Medical Molecular Biology, Beijing Institute of Biotechnology, Collaborative Innovation Center for Cancer Medicine, 100850 Beijing, China; 2 The Third Medical Center of Chinese People’s Liberation Army General Hospital, Beijing, China; 3 The Brain Science Center, Beijing Institute of Basic Medical Sciences, Beijing, China; 4 The First Medical Center of Chinese People’s Liberation Army General Hospital, Beijing, China; 5 Beijing Fengtai District Maternal and Child Health Service Center, Beijing, China; 6 Affiliated Hospital of Qingdao University, Qingdao, China

**Keywords:** Cancer stem cells, Prognostic markers

## Abstract

A further understanding of tumor angiogenesis is urgently needed due to the limited therapeutic efficacy of anti-angiogenesis agents. However, the origin of endothelial cells (EC) in tumors remains widely elusive and controversial. Snail has been thoroughly elucidated as a master regulator of the epithelial–mesenchymal transition (EMT), but its role in endothelium generation is not yet established. In this study, we reported a new and unexpected function of Snail in endothelium generation by breast cancer cells. We showed that high Snail-expressing breast cancer cells isolated from patients showed more endothelium generated from these cells. Expression of Snail was positively correlated with endothelial markers in breast cancer patients. The ectopic expression of Snail induced endothelial marker expression, tube formation and DiI-AcLDL uptake of breast cancer cells in vitro, and enhanced tumor growth and microvessel density in vivo. Snail-mediated endothelium generation depended on VEGF and Sox2. Mechanistically, Snail promoted the expression of VEGF and Sox2 through recruiting the p300 activator complex to these promoters. We showed the dual function of Snail in tumor initiation and angiogenesis in vivo and in vitro through activation of Sox2 and VEGF, suggesting Snail may be an ideal target for cancer therapy.

## Introduction

Tumor angiogenesis is a crucial step for tumor growth, progression, and metastasis. Extensive neovascularization is considered as a major pathological hallmark of cancer. As the major regulator of angiogenesis, the vascular endothelial growth factor (VEGF) pathway represents an ideal therapeutic target for cancer therapy^1,2^. However, a great number of patients have intrinsic resistance or develop acquired resistance to anti-VEGF signaling therapy, suggesting that targeting VEGF signaling alone is not sufficient for effective tumor therapy^3^. Therefore, further understanding of tumor angiogenesis has direct translational implications.

Endothelial cells (EC) are basic components of blood-vessel walls, which are essential in the process of angiogenesis including initiation of vessel sprouting and vessel maturation^4,5^. Previously, it was thought that tumor blood vessels originate from nearby normal vessels or by recruiting circulating endothelial and other cells into the tumor^6,7^. However, increasing researches proposed that cancer stem cell-like (CSC) cells in breast cancer, ovarian cancer, and glioblastomas have the potential to differentiate into EC^8–11^. Recently, it was reported that not only CSCs, but also tumor cells, have the potential to give rise to endothelial phenotypes and directly participate in tumor angiogenesis. Twist1 induces head and neck cancer (HNC) cells to convert to EC through the Jagged1-KLF4 axis^12^. LMO2 mediates EC conversion of glioblastoma cells through acquisition of GSC phenotypes^13^. Further investigation of whether there are additional factors promoting tumor cells into EC lays the foundation for a better understanding of the origin of vascular compartments and vessel maintenance in tumors.

The epithelial–mesenchymal transition (EMT) leads to the migratory phenotype and enhances the tumor-initiating capacity of cancer cells^14^. The transcription factor Snail is a master regulator of EMT^15^, and promotes CSC characteristics in various tumors^16,17^. Snail is well-known as a transcriptional repressor, and directly or indirectly represses the transcription of E-cadherin via recruiting the corepressor complex HDAC1/HDAC2/Sin3A to the 5′-CANNTG-3′ sequence to the E-cadherin promoter^18,19^. Accumulating studies indicate that Snail also acts as a transcriptional activator^20^. However, the mechanism of Snail activating the stemness-related genes and enhancing CSC characteristics is still unknown.

In this study, using genome wide RNA-sequencing analysis, we found Snail regulated the expression of multiple angiogenesis-related genes. Importantly, high Snail-expressing breast cancer cells isolated from patients showed more EC differentiated from these cells. Overexpression of Snail induced endothelium generation of breast cancer cells in a Sox2-dependent and VEGF-dependent manner. Therefore, Snail enhanced tumor progression not only through its tumor-initiating capacity, but also through its ability to promote angiogenesis, suggesting that it may be a promising target for cancer therapy.

## Results

### Snail regulates the expression of multiple angiogenesis-related genes and promotes vascular endothelium generation by breast cancer patients-derived cells in vivo

**Fig. 1:**
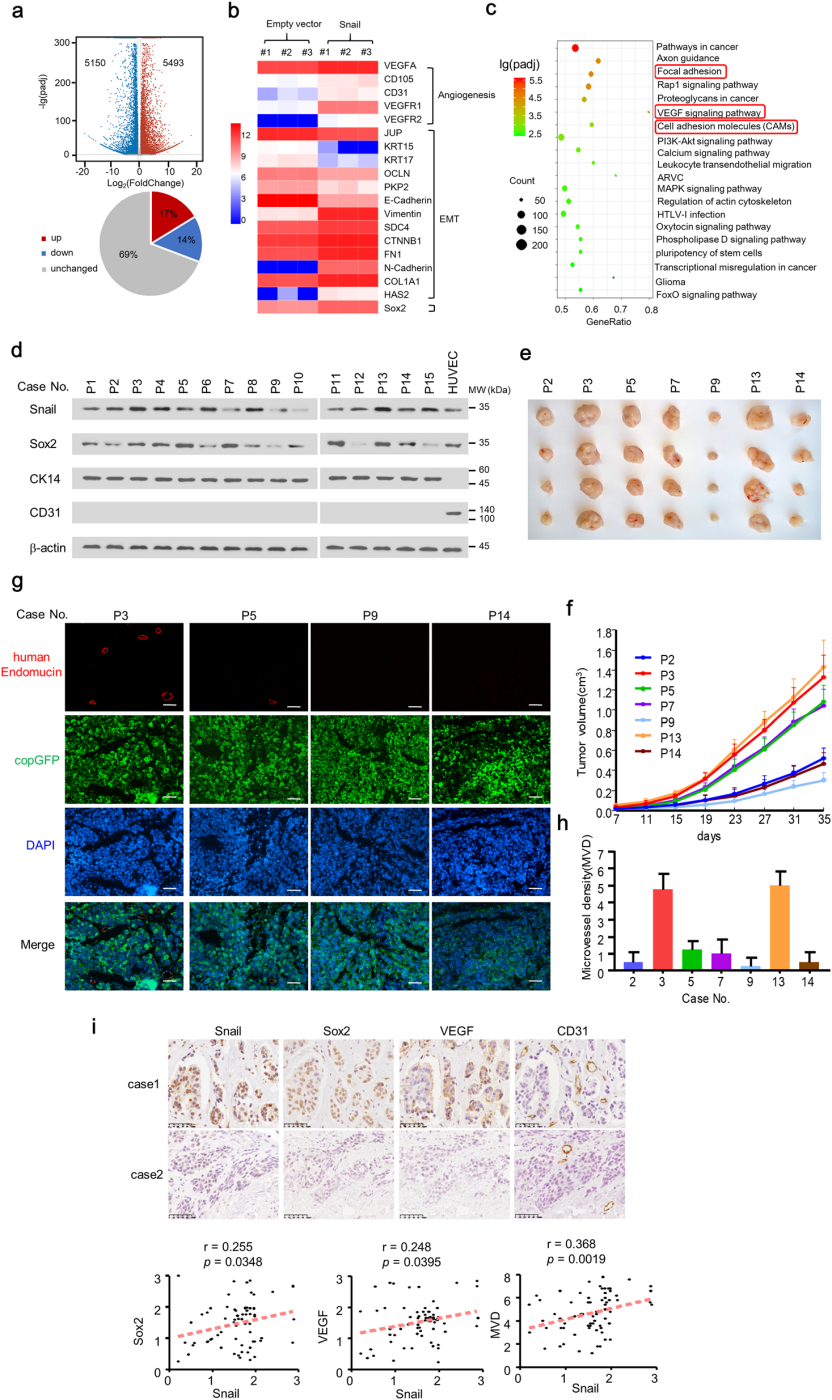
Snail is associated with the angiogenesis pathway and enhances vascular endothelium generation of breast cancer patient-derived cells in vivo. **a** Volcano plot analysis of transcript expression by RNA-seq of ZR75-1 cells stably infected with lentivirus carrying EV or Snail. Transcripts in red were significantly upregulated and transcripts in green were significantly downregulated (fold-change >2.0 and *P* < 0.05). **b** Heatmap of Snail target genes identified by RNA-seq in ZR75-1 cells stably infected with lentivirus carrying EV or Snail. **c** KEGG pathway analysis of genes differentially expressed in ZR75-1 cells stably infected with lentivirus carrying EV or Snail. **d** Representative western blot of Snail and Sox2 in 15 cancerous breast tissues and identification of CK14 and CD31 in isolated breast cancer cells. The HUVEC cells was used as control. The isolated breast cancer cells were labelled with copGFP. **e** Xenograft tumors were established using the isolated breast cancer cells as mentioned in **d**. **f** The tumor growth curves were plotted of **e**, which were measured by vernier caliper at the indicated times. Tumor volumes are presented as means ± SD (*n* = 4). **g** Representative IF staining of xenograft tumors from **e** with anti-human endothelium marker endomucin (red) and copGFP (green). **h** Relative human cell-derived microvessel density (MVD) of xenograft tumor from **g**. **i** Representative immunohistochemical staining of Snail, Sox2, VEGF, and CD31 in 69 cancerous breast tissues. Scale bar: 50 μm. The relationship between Snail and Sox2, VEGF, or MVD was detected by Spearman rank correlation analysis in 69 breast cancer samples. Symbols represent individual samples. *P < 0.05.

To identify downstream effectors of Snail, we performed genome-wide RNA sequencing (RNA-seq) in ZR75-1 cells stably expressing Snail or negative control cells. First, we determined Snail expression in breast cancer cell lines (Fig. S1a), established stable expression of Snail in low Snail-expressing cell lines (MCF-7 and ZR75-1), and knocked down Snail expression in a high Snail-expressing cell line (MDA-MB-231) (Fig. S1b). The RNA-seq results indicated that among 36,225 transcripts detected, 5943 transcripts (17%) were upregulated, and 5150 transcripts (14%) were downregulated (Fig. 1a). Consistent with previous results, Snail inhibited the expression of epithelial markers, such as E-cadherin, KRT15 and KRT18, and promoted the expression of mesenchymal markers, Vimentin and N-cadherin. Unexpectedly, ectopic expression of Snail enhanced the expression of VEGFA and endothelial markers, CD105, CD31, VEGFR1 and VEGFR2 (Fig. 1b). KEGG analysis indicated that Snail regulated the VEGF pathway (Fig. 1c). We further validated EMT, tumor initiation-related genes, and angiogenesis-related genes by qRT-PCR in MCF-7 and ZR75-1 cells (Fig. S1c).

Since Snail overexpression greatly enhanced the expression of VEGF and endothelium markers, we investigated the potential capacity of Snail in differentiating breast cancer cells to EC in vivo. Breast cancer cells were isolated from 15 breast cancer patients and validated with epithelial marker (CK14) and without endothelial marker (CD31) (Fig. 1d). On the other hand, the endothelial cells (HUVEC) expressed endothelial marker (CD31) but not epithelial marker (CK14). The detailed information of breast cancer specimens is presented in Table S1. Seven of 15 breast cancer cell lines isolated from patients successfully developed tumors in NOD-SCID mice (Fig. 1e, f). Moreover, high Snail and Sox2 expression markedly promoted breast tumor growth (Fig. 1d–f). The isolated breast cancer cells were then labelled with copepod super green fluorescent protein (copGFP) to perform cell lineage tracing in nude mice (Fig. 1g). More importantly, compared with low Snail-expressing breast cancer cells (P2, P9 and P14), high Snail-expressing breast cancer cells isolated from patients (P3 and P13) showed more EC differentiated from these cells in seven breast cancer tissues (Fig. 1g, h). Knockdown of Snail in P3 cells decreased the trans-differentiation of breast cancer cells to EC, whereas overexpression of Snail in P9 and P14 cells enhanced the trans-differentiation (Fig. S1d). Furthermore, IHC assay of the 15 breast cancer samples indicated that the expression of Snail positively correlated with microvessel density (MVD) in the original site (Fig. S1e). The specificity of the Snail, Sox2 and VEGF antibodies was confirmed^21^ (Fig. S1f).

Since Snail promoted the expression of the master tumor initiation gene Sox2 (Fig. 1a and c), we also detected whether Sox2 regulated the endothelium generation of breast cancer cells, and found high Sox2-expression modestly induced the generation of breast cancer cells (Fig. 1g, h). We further analyzed the correlation of Snail with Sox2, VEGF, and MVD in breast cancer patients by IHC. Tumors with high Snail and Sox2 expression had significantly greater microvessel number than those with low Snail and Sox2 expression (Fig. 1i). We further validated the correlation between Snail and Sox2 or angiogenesis-related genes expression in breast cancer patients from the cancer genome atlas (TCGA) and GEO database (NCBI Gene Expression Omnibus) (Table S2). Moreover, samples both in the GEO database and we collected with high Snail or Sox2 expression had shorter disease-free survival (DFS) and overall survival (OS) (Fig. S1e). Snail/Sox2 expression was positively associated with tumor size, nodal status, and grade, but they were not associated with the expression of estrogen receptor *α* (ER*α*), progesterone receptor (PR), age, or human epidermal growth factor receptor 2 (HER2) (Table S3). With univariate analysis by Cox proportional hazards model, tumor size, nodal status, grade, ER, PR, Snail and Sox2 status were demonstrated as significant prognostic parameters for DFS and OS (Table S4). Taken together, these results indicated that Snail is essential in breast cancer progression through regulating endothelium generation.

### Snail induces EC generation of breast cancer cells in vitro

**Fig. 2:**
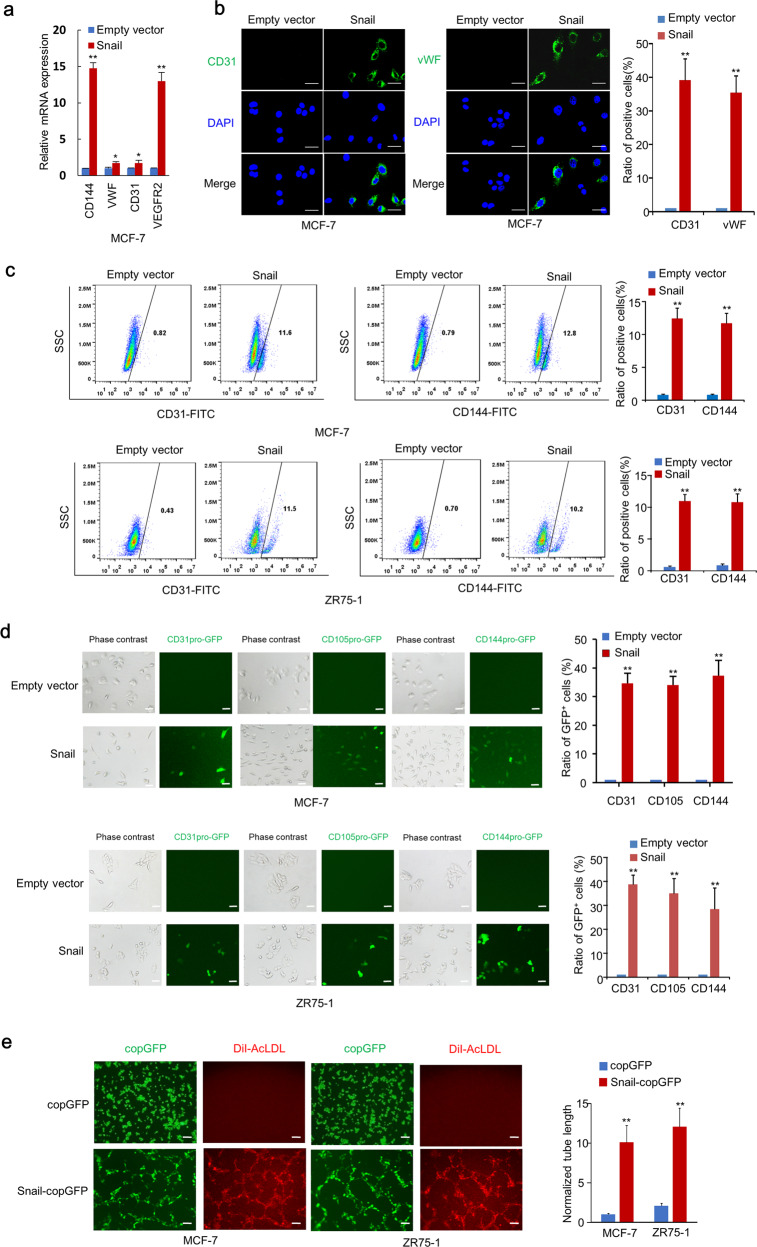
Snail induces endothelium generation of breast cancer cells in vitro. **a** Relative mRNA expression of endothelium markers in MCF-7 cells stably infected with lentivirus carrying EV or Snail was determined by qRT-PCR assay. **b** Representative confocal images of MCF-7 cells stably infected with lentivirus carrying EV or Snail labelled with endothelium markers CD31 or vWF. The nuclei were stained with DAPI (blue). Scale bar, 20 μm. Graphs show the percentage of endothelium marker-positive cells. Results shown are mean ± SD of three independent experiments. **c** Representative FACS with the indicated antibodies in MCF-7 and ZR75-1 cells stably infected with EV or Snail. Statistical analyses of CD31-positive or CD144-positive rates are shown in the right panel. Results shown are mean ± SD of three independent experiments. **d** Endothelium lineage tracing of MCF-7 and ZR75-1 cells stably infected with EV or Snail using CD31 promoter-driven GFP (CD31pro-GFP), CD105 promoter-driven GFP (CD105pro-GFP), or CD144 promoter-driven GFP (CD144pro-GFP). Scale bar, 5 μm. Statistical analysis of GFP-positive rates is shown in the right panel. Results shown are mean ± SD of three independent experiments. **e** Tube-forming assays and DiI-AcLDL uptake assays with Snail-copGFP-expressing or copGFP-expressing MCF-7 and ZR75-1 cells. Statistical analysis of tube length is shown in the right panel. Results shown are mean ± SD of three independent experiments. Scale bar, 100 μm. Red: DiI-AcLDL staining. *P < 0.05, **P < 0.01.

Since Snail enhanced the expression of multiple genes in the angiogenesis pathway and differentiated breast cancer cells into endothelium cells in vivo, we further investigated whether ectopic expression of Snail regulated generation of breast cancer cells into EC in vitro. Snail overexpression improved the mRNA expression of endothelial markers, such as CD144 (VE-cadherin), vWF, CD31, and VEGFR2, in MCF-7 and ZR75-1 cells, whereas knockdown of Snail in MDA-MB-231 cells decreased the mRNA expression of endothelial markers (Figs. 2a and S2a). Immunofluorescent (IF) staining showed that the endothelial markers in MCF-7 and ZR75-1 cells were expressed in ~30% of Snail-overexpressing cells, and flow cytometry analysis showed ~10% (Figs. 2b, c and S2b). To further determine the capacity of Snail to transdifferentiate breast cancer cells to EC, we performed cell lineage tracing of Snail overexpressing cells with endothelial marker, including CD31, CD105, and CD144 promoter-driven expression of GFP, which served as fluorescent reporters of endothelial lineage. We confirmed that the CD31, CD105, and CD144 promoters were functional and endothelial cell-specific, since CD31, CD105, and CD144 promoter-driven GFP expression occurred specifically in EC (HUVEC), but not in epithelial cells (MCF-7 and ZR75-1 cells) (Fig. S2c). We found that ~30% of Snail-overexpressing MCF-7 and ZR75-1 cells were GFP-positive EC. In contrast, the negative control cells had no GFP-positive cells (Fig. 2d). It is interesting to note that 10% of MDA-MB-231 cells are GFP-positive EC, and MDA-MB-231 cells with Snail knock-down had no GFP-positive EC (Fig. S2d). Snail-overexpressing MCF-7 and ZR75-1 breast cancer cells demonstrated increased tube-forming ability and DiI-acetylated low-density lipoproteins (AcLDL) uptake capability (Fig. 2e). Consistent with the above results, knockdown of Snail in MDA-MB-231 cells decreased tube-forming ability (Fig. S2e). These data indicated that Snail regulates the formation of EC from breast cancer cells.

### Snail induces stemness properties and endothelial generation of breast cancer cells through Sox2

**Fig. 3:**
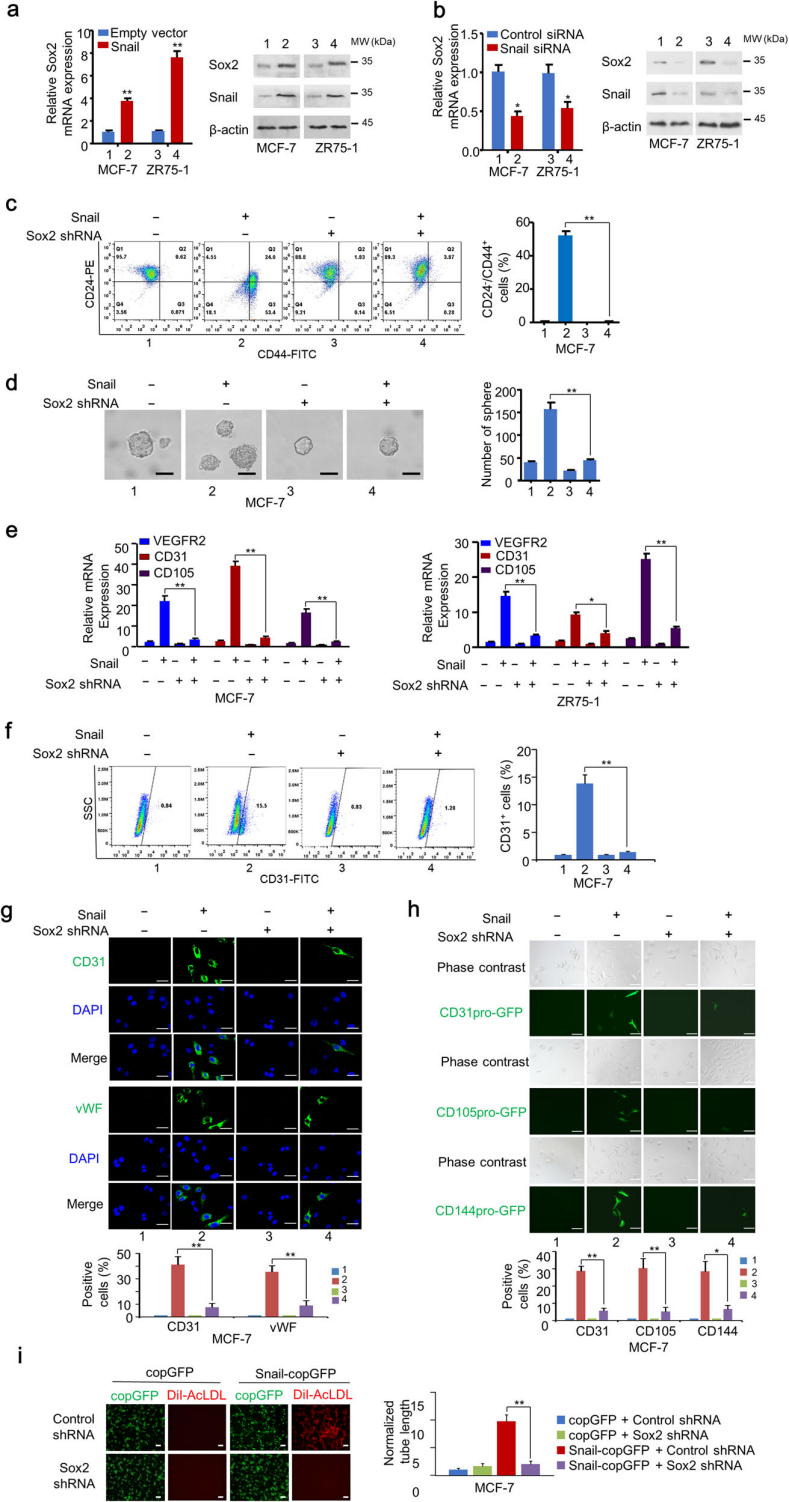
Snail induces stemness properties and endothelium generation of breast cancer cells through Sox2. **a** qRT-PCR analysis of relative mRNA expression of Sox2 in MCF-7 and ZR75-1 cells stably infected with lentivirus carrying EV or Snail. **b** qRT-PCR analysis of relative mRNA expression of Sox2 in MCF-7 and ZR75-1 cells transfected with control siRNA or Snail siRNA. **c** Representative FACS of MCF-7 cells infected with the indicated plasmids CD44-FITC and CD24-PE. Statistical analysis of CD24^−^/CD44^+^ rates is shown in the right panel. Results shown are mean ± SD of three independent experiments. **d** Mammosphere assay of MCF-7 cells infected with indicated plasmids. Graphs show the relative number of mammospheres. Results shown are mean ± SD of three independent experiments. Scale bar, 20 μm. **e** qRT-PCR analysis of relative mRNA expression of endothelium markers VEGFR2, CD31, and CD105 in MCF-7 and ZR75-1 cells stably infected with the indicated plasmids. **f** FACS analysis of MCF-7 cells infected with indicated plasmids using CD31-FITC antibodies. Statistical analysis of CD31^+^ rates is shown in the right panel. Results shown are mean ± SD of three independent experiments. **g** Representative confocal images of MCF-7 cells infected with the indicated plasmids labelled with endothelium markers CD31 and VWF. The nuclei were stained with DAPI (blue). Scale bar, 20 μm. Graphs show the percentage of endothelium marker-positive cells. Results shown are mean ± SD of three independent experiments. **h** Lineage tracing of MCF-7 cells stably infected with EV or Snail using CD31pro-GFP, CD105pro-GFP, or CD144pro-GFP. Scale bar, 10 μm. Graphs show the percentage of GFP-positive cells. Results shown are mean ± SD of three independent experiments. **i** Tube-forming assays and DiI-AcLDL uptake assays with Snail-copGFP-expressing or copGFP-expressing MCF-7 cells. Statistical analysis of tube length is shown in the right panel. Results shown are mean ± SD of three independent experiments. Scale bar, 100 μm. Red: DiI-AcLDL staining. *P < 0.05, **P < 0.01.

Since EMT generates cells with stem-like properties, and Snail enhanced critical stemness-related Sox2 expression, we investigated the effect of Sox2 on Snail-mediated stem-like properties and endothelium generation. We first confirmed the EMT phenotype of MCF-7 and ZR75-1 cells stably expressing Snail. As expected, overexpression of Snail greatly inhibited the expression of E-cadherin and promoted the expression of N-cadherin (Fig. S3a). Western blotting and qRT-PCR analysis further confirmed that overexpression of Snail increased Sox2 mRNA and protein levels in MCF-7 and ZR75-1 cells, while the knockdown of Snail decreased Sox2 mRNA and protein levels (Fig. 3a, b). CD44^high^/CD24^low^ and ALDH1-high cells are considered as breast CSCs. Snail overexpression greatly enhanced the CD44^high^/CD24^low^ and ALDH1-high population in MCF-7 and ZR75-1 cells, while Sox2 knockdown abolished this effect, suggesting Snail triggered CSC-like population enhancement through Sox2 (Figs. 3c and S3b). Mammosphere formation assay also demonstrated that Snail mediated CSC sphere formation through Sox2 (Fig. 3d, Fig. S3c). These results indicated the essential role of Sox2 in Snail-mediated CSC-like phenotypes. Since Snail transdifferentiated breast cancer cells into EC, and triggered cancer stem-like phenotypes through Sox2, we investigated the role of Sox2 in Snail-mediated generation of breast cancer cells into EC. qRT-PCR analysis showed increased expression of endothelial markers, such as VEGFR2, CD31, and CD105 in Snail-overexpressing MCF-7 and ZR75-1 cells. However, knockdown of Sox2 almost abolished this effect (Fig. 3e). Flow cytometry and IF staining analysis further validated the effect of Sox2 (Figs. 3f, g and S3d, e). Sox2 knockdown abolished Snail-inducing endothelial cell marker promoter activity, tube-forming ability, and DiI-AcLDL uptake ability (Figs. 3h, i and S3f, g). To summarize, Snail-mediated breast cancer cell stem-like phenotypes and endothelium generation is dependent on Sox2.

Since E-cadherin is reported to correlate with angiogenesis, we detected the effect of E-cadherin on Snail-mediated angiogenesis. Enforced expression of E-cadherin in Snail-overexpressing MCF-7 and ZR75-1 cells did not affect Snail-induced tube-forming and DiI-AcLDL uptake ability (Fig. S4a, b). Furthermore, lineage tracing analysis indicated that E-cadherin had no effect on Snail-mediated trans-differentiation of epithelial cells into EC and blood vessels formation in vivo (Fig. S4c). In conclusion, Snail-mediated angiogenesis did not depend on E-cadherin.

### Sox2 fails to induce endothelium generation of breast cancer cells

**Fig. 4:**
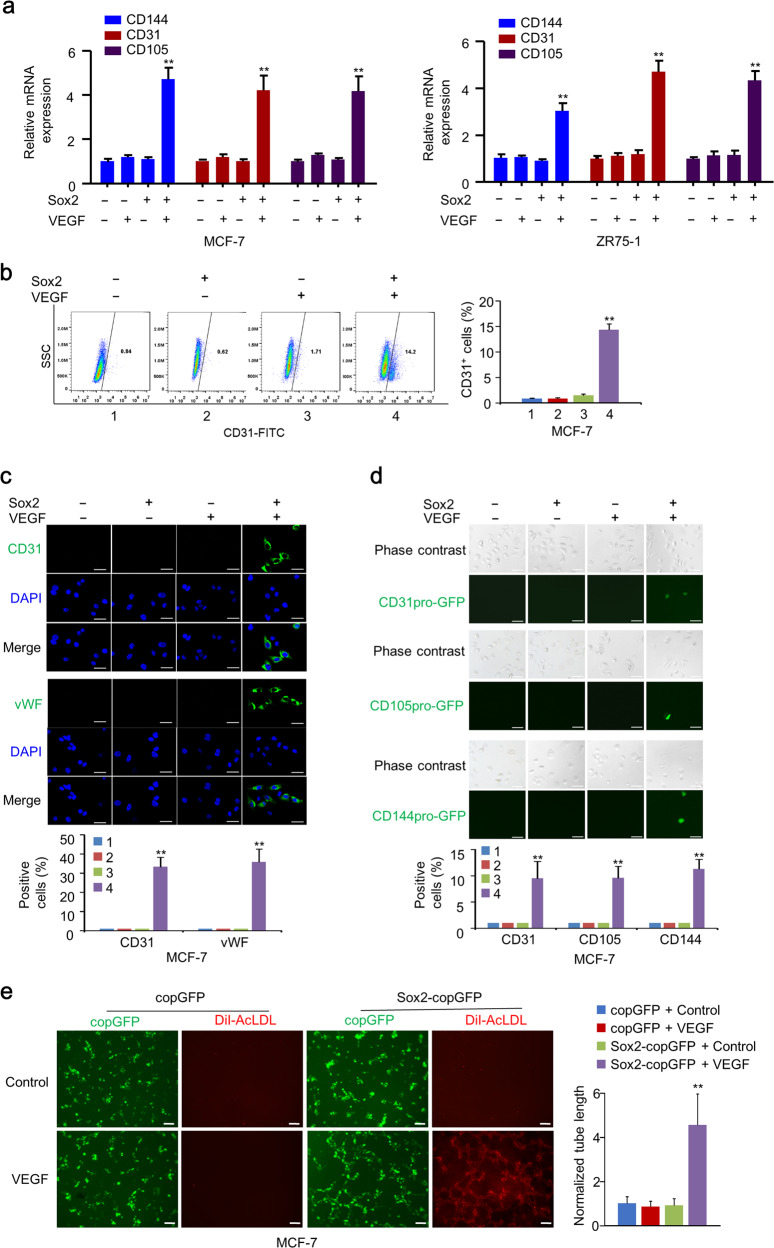
Sox2 alone fails to induce endothelium generation in breast cancer cells. **a** qRT-PCR analysis of endothelium markers CD144, CD31, and CD105 in EV-stably or Sox2-stably expressing MCF-7 and ZR75-1 cells cultured with or without VEGF. **b** FACS analysis with CD31-FITC in EV-stably or Sox2-stably expressing MCF-7 and ZR75-1 cells cultured with or without VEGF. Statistical analysis of CD31^+^ rates is shown in the right panel. Results shown are mean ± SD of three independent experiments. **c** IF assays of MCF-7 cells infected with lentivirus carrying Sox2 or EV and cultured with or without VEGF using endothelium markers CD31 and VWF. The nuclei were stained with DAPI (blue). Scale bar, 20 μm. The graphs show the percentage of endothelium marker-positive cells. Results shown are mean ± SD of three independent experiments. **d** Lineage tracing of EV-stably or Sox2-stably expressing MCF-7 cells with CD31pro-GFP, CD105pro-GFP, or CD144pro-GFP cultured with or without VEGF. Scale bar, 10 μm. Graphs show percentage of GFP-positive cells. Results shown are mean ± SD of three independent experiments. **e** Tube-forming assays and DiI-AcLDL uptake assays with copGFP-overexpressing or Sox2-copGFP-overexpressimg MCF-7 cells cultured with or without VEGF. Statistical analysis of tube length is shown in the right panel. Results shown are mean ± SD of three independent experiments. Scale bar, 100 μm. Red: DiI-AcLDL staining. *P < 0.05, **P < 0.01.

Next, we detected whether Sox2 promoted endothelial cell generation from breast cancer cells. qRT-PCR, flow cytometry, and IF analyses indicated that Sox2 alone could not induce endothelial marker expression in MCF-7 and ZR75-1 cells (Figs. 4a–c and S5a, b). However, Sox2 overexpression in MCF-7 and ZR75-1 cells treated with VEGF induced the expression of endothelial cell markers (Figs. 4a–c and S5a, b). In addition, Sox2-overexpressing MCF-7 and ZR75-1 cells stimulated with VEGF enhanced endothelial marker promoter activity, tube-forming ability, and DiI-AcLDL uptake, but Sox2 overexpression or VEGF stimulation alone could not (Figs. 4d, e and S5c, d).

### Endothelium generation of breast cancer cells induced by Snail depends on VEGF signaling

**Fig. 5:**
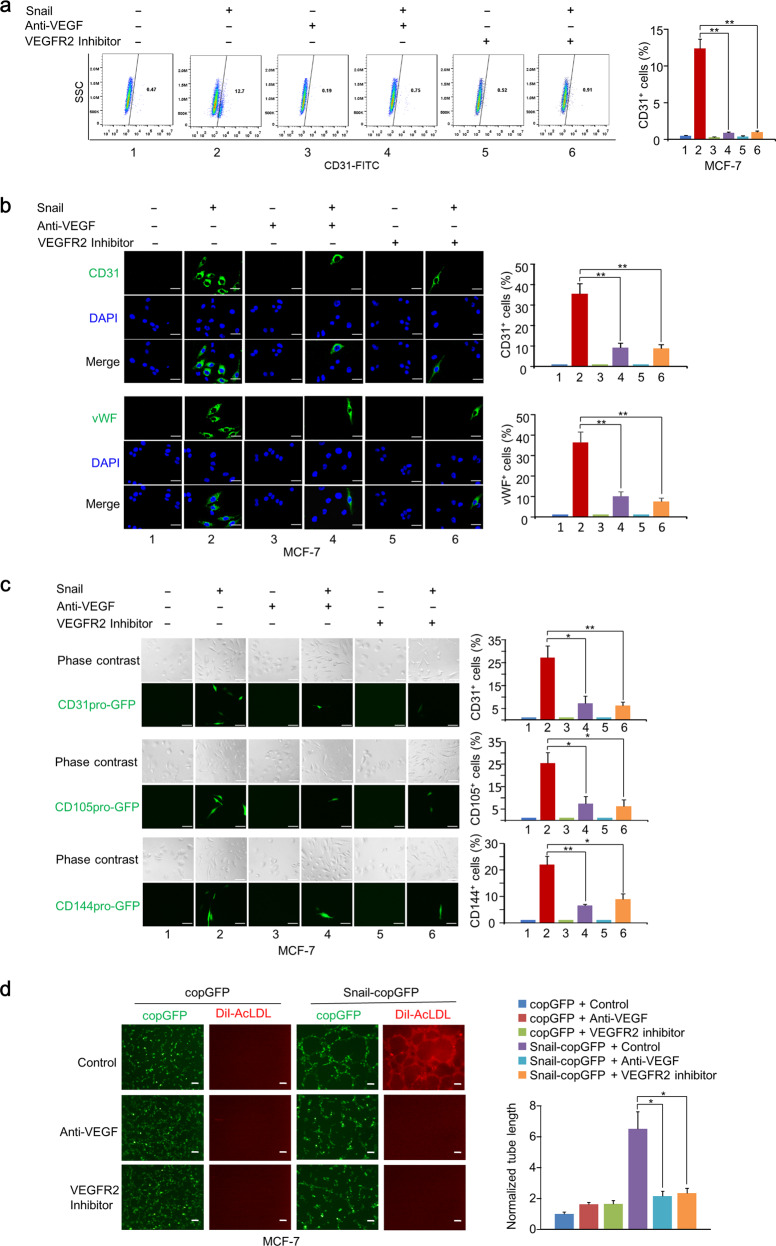
Endothelium generation of breast cancer cells induced by Snail depends on VEGF signaling. **a** Representative FACS of MCF-7 cells stably expressing EV or Snail treated with or without VEGF-neutralized antibody or VEGFR2 inhibitor using CD31-FITC antibodies. Graphs show percentage of CD31-positive cells. Results shown are mean ± SD of three independent experiments. **b** IF analysis of MCF-7 cells stably expressing EV or Snail treated with or without VEGF neutralized antibody or VEGFR2 inhibitor. Graphs show percentage of CD31-positive or VWF-positive cells. Results shown are mean ± SD of three independent experiments. Scale bar, 20 μm. **c** Lineage tracing of MCF-7 cells stably expressing EV or Snail with CD31pro-GFP, CD105pro-GFP, and CD144pro-GFP treated with or without VEGF-neutralized antibody or VEGFR2 inhibitor. Scale bar, 10 μm. Graphs show percentage of GFP-positive cells. Results shown are mean ± SD of three independent experiments. **d** Tube-forming assays and DiI-AcLDL uptake assays with copGFP-expressing or Snail-copGFP-expressing MCF-7 cells treated with or without VEGF-neutralized antibody or VEGFR2 inhibitor. Statistical analysis of tube length is shown in the right panel. Results shown are mean ± SD of three independent experiments. Scale bar, 100 μm. Red: DiI-AcLDL staining. *P < 0.05, **P < 0.01.

The observation that Sox2 or VEGF alone failed to induce the endothelium generation of breast cancer cells prompted us to determine the role of VEGF signaling in this process. We evaluated the effect of VEGF-neutralized antibody and VEGF-receptor inhibitor, both anti-angiogenic drugs, on the generation of Snail-induced EC in MCF-7 and ZR75-1 cells. Indeed, we found that both VEGF-neutralized antibody and VEGF-receptor inhibitor abolished Snail-mediated endothelial marker expression, as determined by flow cytometry and IF analysis (Figs. 5a, b and S6a, b). Moreover, the blockade of VEGF signaling greatly impaired Snail-induced endothelial marker promoter activity, tube-forming ability, and DiI-AcLDL uptake capability (Fig. 5c, d and S6c, d). Taken together, endothelial generation of breast cancer cells induced by Snail is dependent on VEGF signaling.

### Snail and p300 co-activate Sox2 and VEGF transcription

**Fig. 6:**
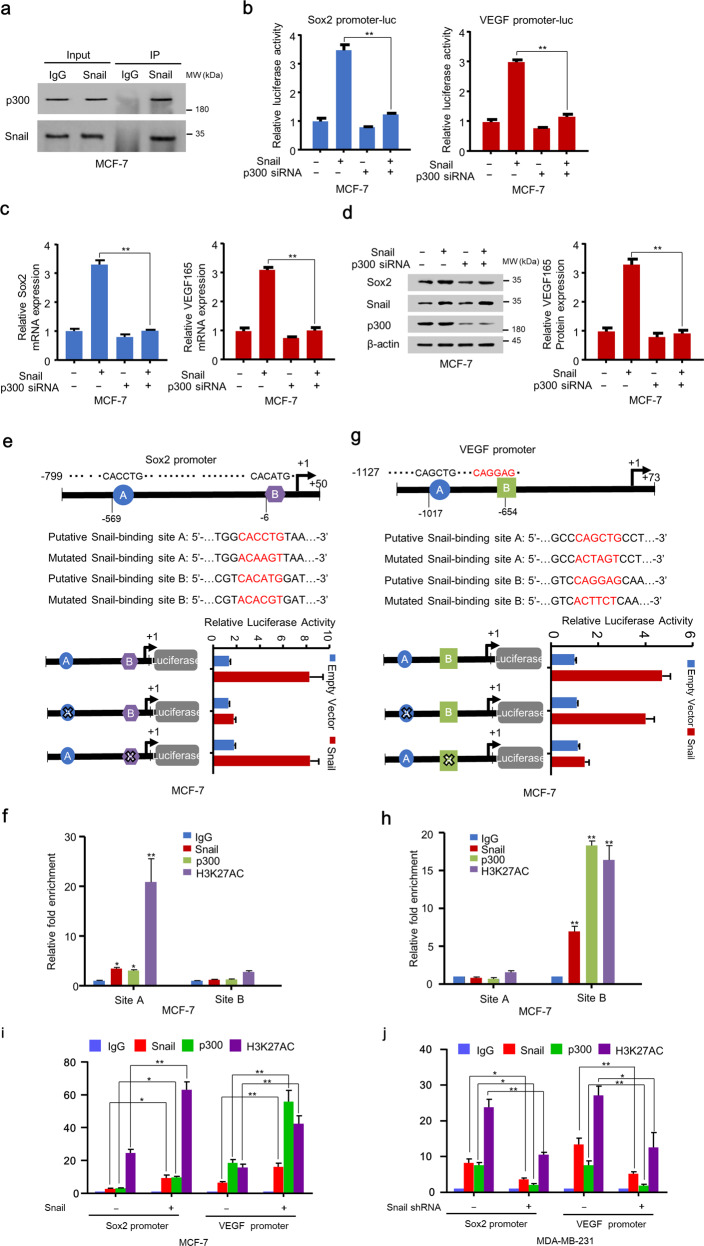
Snail and p300 co-activate Sox2 and VEGF transcription. **a** MCF-7 cells were immunoprecipitated with Snail antibodies or pre-immune control serum (IgG) followed by western blotting with antibodies against p300 or Snail. **b** Luciferase reporter assay of MCF-7 cells cotransfected with Sox2 promotor-Luc or VEGF promotor-Luc with control siRNA or p300 siRNA. **c** qRT-PCR analysis of Sox2 or VEGF_165_ mRNA expression in MCF-7 and ZR75-1 cells transfected with the indicated plasmids. **d** Western blotting and ELISA of MCF-7 cells transfected with the indicated plasmids using the indicated antibodies. **e** Relative luciferase activity of wild-type and mutated Sox2 promotor reporter constructs in MCF-7 cells transfected with empty vector or Snail. A and B indicate putative binding sites of Snail. The ‘X’ symbol denotes a mutated Snail-binding site. **f** ChIP analysis with the indicated antibodies in MCF-7 cells showed the occupancy of Snail, p300, and H3K27AC protein on putative Snail-binding sites in the Sox2 promoter. All values shown are means ± SD of three independent experiments performed in triplicate. **g** Relative luciferase activity of wild-type and mutated VEGF promotor reporter constructs in MCF-7 cells transfected with empty vector or Snail. A and B indicate putative binding sites of Snail. The ‘X’ symbol denotes a mutated Snail-binding site. **h** ChIP analysis with the indicated antibodies in MCF-7 cells showed the occupancy of Snail, p300, and H3K27AC protein on putative Snail-binding sites in the VEGF promoter. All values shown are means ± SD of three independent experiments performed in triplicate. *P < 0.05, **P < 0.01. **i** ChIP analysis with the indicated antibodies in MCF-7 cells stably infected with lentivirus carrying EV or Snail showed the occupancy of Snail, p300, and H3K27AC proteins on Snail-binding sites in the Sox2 and VEGF promoters. **j** ChIP analysis with the indicated antibodies in MDA-MB-231 cells infected with lentivirus carrying negative control or Snail shRNA indicated the occupancy of Snail, p300, and H3K27AC proteins on Snail-binding sites in the Sox2 and VEGF promoters. All values shown are means ± SD of three independent experiments performed in triplicate. *P < 0.05, **P < 0.01.

Since Snail ectopic expression enhanced the mRNA expression of VEGF and Sox2 (Figs. 1b and S1c), we further investigated the mechanism of Snail regulating the expression of VEGF and Sox2. It has been reported that Snail interacted with acetyltransferase p300/CBP to activate the transcription of target genes^20^. We first confirmed the interaction between p300 and Snail in MCF-7 cells (Fig. 6a). Furthermore, Snail overexpression enhanced the transcriptional activity of VEGF and Sox2 promoter, while p300 knockdown almost abolished Snail-mediated enhancement of transcriptional activity of VEGF and Sox2 promoter (Fig. 6b). Consistent with the luciferase reporter assay, Snail enhanced the mRNA and protein expression of VEGF and Sox2, whereas p300 knockdown counteracted this effect (Fig. 6c, d). These results demonstrated that activation of VEGF and Sox2 transcription by Snail was dependent on p300. Since Snail promoted the transcriptional activity of the Sox2 and VEGF promoters, we then determined the binding site of Snail on the Sox2 and VEGF promoters. We found two putative binding sites of Snail on the Sox2 (from −799 to +50 bp) and VEGF promoters (from −1127 to +73 bp) (Fig. 6e, g). Mutated promoter reporter analysis was performed to determine the binding site of Snail on the Sox2 and VEGF promoters. Snail increased the Sox2 promoter activity of the reporter with mutated site B, but not with mutated site A in breast cancer cells, suggesting Snail bound to site A to increase Sox2 promoter reporter activity (Fig. 6e). In contrast, site B of the VEGF promoter was responsible for Snail modulation of VEGF promoter activity, since Snail increased the activity of the reporter with mutated site A, but not with mutated site B in breast cancer cells (Fig. 6g). Furthermore, chromatin immunoprecipitation (ChIP) assay showed that Snail, p300, and H3K27AC coactivator complex were recruited to the region containing site A of the Sox2 promoter, but not the region containing site B, and the coactivator complex was recruited to site B of the VEGF promoter, but not to the site B sequence (Fig. 6f, h). Overexpression of Snail promoted the recruitment of p300 and H3K27AC on Sox2 (site A) and VEGF (site B) promoters in MCF-7 cells, and knockdown of Snail inhibited the recruitment of p300 and H3K27AC on the promoters in MDA-MB-231 cells (Fig. 6i, j). Taken together, these data suggest that the Snail/p300/H3K27AC coactivator complex binds to the Sox2 and VEGF promoters to increase their transcription.

### Snail induced stem-like properties and endothelial cell generation of breast cancer cells in vivo

**Fig. 7:**
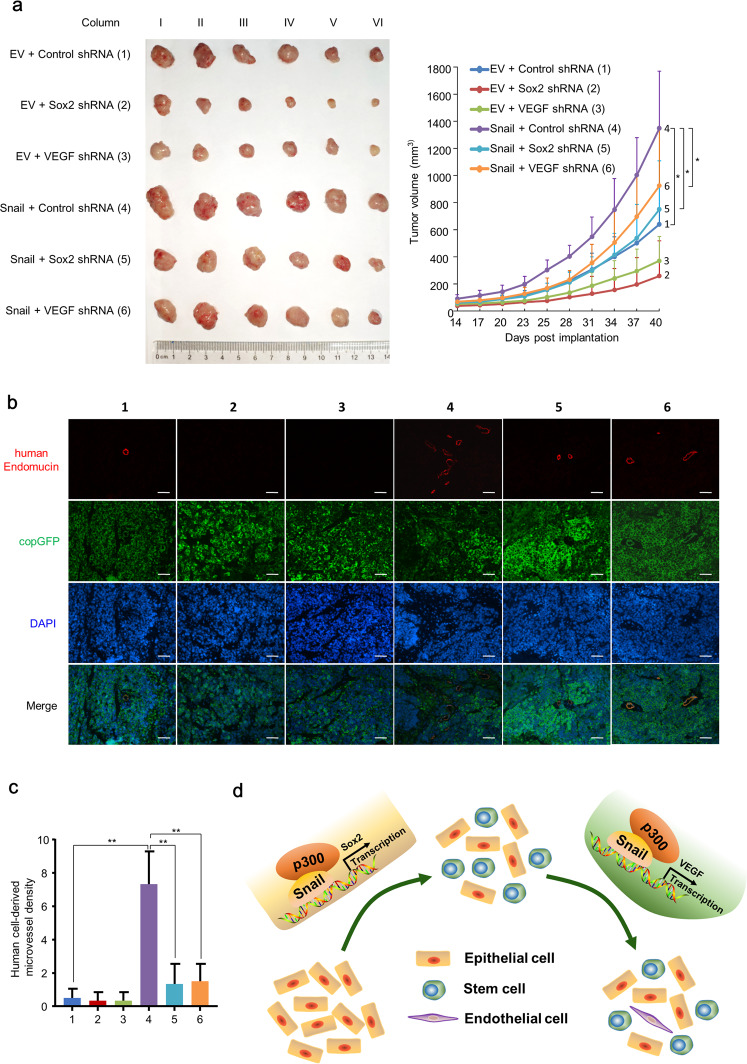
Snail promotes tumor growth and endothelium generation in breast cancer cells in vivo. **a** Volume of xenograft tumors of ZR75-1 cells infected with lentivirus carrying Snail-copGFP or copGFP and Sox2 shRNA or VEGF shRNA. The tumors were measured by vernier caliper and tumor growth curves were plotted. Tumor volumes are presented as means ± SD (*n* = 6). **b** Representative IF staining of endothelium marker endomucin and copGFP in xenograft tumors of the indicated groups. Scale bar: 50 μm. **c** Graphs show relative MVD of the indicated groups. Data are shown as means ± SD. **d** Graphic summary of Snail promoting tumor initiation and endothelium cell differentiation of breast cancer cells. Snail recruits p300 activation complex to the Sox2 promoter and enhances the cancer stem cell properties. Meanwhile, Snail/p300 complex binds to the VEGF promoter and activates the expression of VEGF. Elevated VEGF expression enhances the generation of breast CSCs to endothelial cells. *P < 0.05, **P < 0.01.

Next, we further determined the capability of Snail-induced endothelial generation of breast cancer cells in vivo using tumor xenograft models in nude mice. Snail overexpression greatly enhanced tumor growth, whereas Sox2 or VEGF knockdown inhibited breast tumor growth (Fig. 7a). However, Sox2 or VEGF knockdown greatly inhibited Snail-induced increasement of tumor growth (Fig. 7a). By lineage-tracing analysis, we found that Snail-overexpressing breast cancer cells transdifferentiated into EC and formed blood vessels directly in vivo (Fig. 7b). However, knockdown of Sox2 or VEGF counteracted the effect of Snail-mediated endothelial generation by breast cancer cells, indicating that Snail increased blood vessel formation through Sox2 and VEGF (Fig. 7b, c).

## Discussion

Tumor angiogenesis is a hallmark of cancer development, which has been considered as an attractive therapeutic target^3^. At present, many anti-angiogenesis therapies have been developed to target the VEGF pathway^22^. However, this anti-cancer strategy is challenging because of clinical resistance and side effects^2,23^. Recently, accumulating evidences suggest that the pathway of regulating CSC and angiogenesis are closely related, which may provide new targets for cancer therapy^24,25^. In this report, we reveal an unexpected function of Snail in endothelium generation of breast cancer cells. Firstly, Snail regulated the expression of multiple genes of the angiogenesis pathway, and high Snail-expressing breast cancer cells derived from patients transdifferentiated into EC and formed blood vessels directly in vivo, suggesting Snail is essential in the angiogenesis of breast cancer. Secondly, Snail induced the expression of endothelial markers, such as CD144, CD31, and CD105. Approximately 10% of Snail-expressing breast cancer cells acquired endothelial markers, indicating this portion of breast cancer cells had transdifferentiated into EC from epithelial cells. Thirdly, Snail-overexpressing breast cancer cells could form tubes and uptake low-density lipoprotein, and selective targeting of these human vessels diminished the xenograft tumors volume, suggesting Snail-derived endothelial vessels are functional. Taken together, our data suggest that intervention with Snail can inhibit not only angiogenesis, but also the self-renewal of CSC.

It is well established that embryonic stem cells can differentiate into EC dependent on VEGF and its receptors. However, the role of VEGF signaling in CSCs differentiation to EC is controversial. Many studies have indicated that VEGF signaling is involved in CSC generation to EC. Blocking VEGF with the anti-angiogenesis agent bevacizumab or silencing VEGFR2 with shRNA inhibited the differentiation of glioblastoma CSCs into endothelial progenitors^26^. VEGF induces breast CSCs to express endothelial markers in vitro and incorporate into tumor vasculature in vivo^27^. CSCs isolated from human renal carcinomas and poorly differentiated colon adenocarcinoma cells acquired an endothelial phenotype in response to VEGF stimulation^28^. In contrast, Soda et al. demonstrated that GBM-initiating cells are able to differentiate into ECs in a VEGF-independent manner^29^. Furthermore, CSCs of ovarian cancer that have transdifferentiated to ECs are VEGF-independent but IKK*β*-dependent^30^. Here, we show that VEGF blockade with neutralizing antibody or the VEGFR2 inhibitor sunitinib impaired Snail-induced endothelial markers expression and promoter activity, and capillary structure formation in matrigel by breast cancer cells induced by Snail. In addition, knockdown of VEGF inhibited Snail-induced angiogenesis and tumor cell proliferation in nude mice. These results suggested that Snail induced breast cancer cell generation into EC in a VEGF-dependent and VEGFR-dependent manner.

Previously, Snail was known as a transcriptional repressor through recruiting corepressor complexes HDAC1–HDAC2, AJUBA–PRMT5, or PRC2 to target gene promoters^31,32^. Recently, growing evidence suggests that Snail is also a transcriptional activator through recruiting coactivator complexes to target genes^20^. Here, our data indicated that Snail upregulated the transcription of 9040 genes. Further investigation of the mechanism indicated that Snail activated gene transcription by recruiting CBP/p300 to the target gene (Sox2 and VEGF) promoters and enhancing the acetylation of H3K27. In addition, it is the first time that Sox2 and VEGF were identified as target genes of Snail. Sox2 was positively correlated with Snail in breast cancer patients, which is in agreement with several recent studies on esophageal squamous cell carcinoma^33^.

Although the association between EMT and stem-like properties induced by Snail is well established, the underlying mechanisms remain largely unknown. Here, we identified Sox2, an important stemness-related gene, which contributes to Snail-induced stem-like properties. Sox2 knockdown greatly reduced the stemness properties induced by Snail. Moreover, Sox2 knockdown almost abolished Snail pro-proliferation activity in vivo. Our findings elucidated a connection between EMT and CSC properties induced by Snail in breast cancer cells.

It has been reported that increased expression of Sox2 is positively correlated with angiogenic factors in retinoblastoma tissues, implying Sox2 have a role in tumor angiogenesis^34^. Our data indicated that Sox2 alone could not induce the generation of breast cancer cells to EC in vitro, but successfully induced endothelium generation with VEGF. However, high Sox2-expressing breast cancer cells derived from patients modestly differentiated into EC in vivo, which may be due to the secretion of VEGF of cancer cells in vivo. Furthermore, knockdown of Sox2 almost abolished Snail-induced endothelial cell generation by breast cancer cells, suggesting both Sox2-induced breast cancer cell dedifferentiation to CSCs and VEGF-induced differentiation of CSCs to EC are necessary for breast cancer cell transdifferentiation. The dual function of the Snail-enhanced angiogenic factor VEGF and the stemness gene Sox2 may partially explain the dysfunction of anti-angiogenesis agent in these tumors. The complex interaction between angiogenic and stem cell-related pathways in these tumors may be necessary for the development of effective drugs. Therefore, identification of angiogenic and CSC regulators reveals a new approach for targeted cancer therapy.

## Materials and methods

### Cell culture

Human embryonic kidney HEK293T cells and human breast cancer MCF-7, ZR75-1, BT474, SKBR3, T47D, MDA-MB-231, and HUVEC cells were purchased from American Type Culture Collection (ATCC), and have previously been examined for mycoplasma contamination. HUVEC cells were maintained in EGM-Plus BulletKit Endothelial Cell Growth Medium BulletKit (Lonza). HEK293T cells and human breast cancer cells were maintained in DMEM (Invitrogen) containing 10% FBS (Hyclone).

### Isolation of breast cancer patients-derived cells

Tumor specimen was finely minced into 1–2 mm^3^ pieces, and digested through incubation in advanced Dulbecco’s modified Eagle medium (DMEM) containing collagenase II (Sigma) for 1 h at 37 °C. The cell suspension was washed with PBS and then separated from matrix and aggregates through a graded series of meshes, and filtrated through 40 μm pore filter (Millipore). The cells were then incubated in serum-free DMEM-F12 (Gibco) supplemented with 10 ng/ml basic fibroblast growth factor (bFGF), 20 ng/ml epidermal growth factor (EGF), 5 μg/ml insulin, and 0.4% bovine serum albumin (Sigma) at 37 °C in a humidified 5% CO_2_ atmosphere. Breast cancer samples were collected from Chinese PLA General Hospital with the informed consent of patients, and with the approval of the Institutional Review Committees of Chinese PLA General Hospital.

### RNA sequencing

Total RNA was extracted from ZR75-1 cells stably infected with lentivirus carrying EV or Snail by TRIzol reagent according to the manufacturer’s protocol (Invitrogen). RNA integrity was assessed with the RNA Nano 6000 Assay Kit (Agilent Technologies). Sequencing by Illumina HiSeq 2100 platform (Illumina, San Diego, CA, USA) was performed after mRNA purification, mRNA fragmentation, first-strand cDNA synthesis, second-strand cDNA synthesis, library fragments selection (preferentially 200 bp in length), and PCR amplification.

### Plasmids, lentiviruses, siRNAs, and reagents

Lentiviral vectors for Snail and Sox2 were obtained by inserting PCR-amplified gene fragments into pCDH (System Biosciences) or pCDH-copGFP (copepod super GFP, System Biosciences). Human CD144 promoter was obtained by PCR, and inserted into pCDH to replace the original CMV promoter, and ORF of GFP was then inserted into the vector to generate fluorescent reporter. CD105 promoter-GFP and CD31 promoter-GFP were generous gifts from Dr. Shideng Bao (Lerner Research Institute). The Sox2 and VEGF promoter luciferase reporters were obtained by PCR, and inserted into pGL4-basic vector (Promega). The mutated Sox2 and VEGF promoter luciferase reporters were constructed by recombinant PCR. The primers used for promoters and mutations are listed in Table S5. The short hairpin RNA (shRNAs) targeting Sox2 or VEGF were inserted into PSIH-H1-puro (System Bioscience). The small interfering RNAs sharing the same target Snail or p300 were synthetized from GenePharma (Shanghai, China). The sequences for siRNAs and shRNAs are listed in Table S6. Lentiviruses were produced by co-transfecting HEK293T cells with recombinant lentivirus vectors and pPACK Packaging Plasmid Mix (System Biosciences) using Lipofectamine 3000 Reagent (Invitrogen) following the manufacture’s protocols. Viral supernatants were harvested 48 h after transfection. The target cells were then infected with the lentiviral constructs with 8 μg/ml polybrene (Sigma-Aldrich). Stable cell lines were selected in 1 μg/ml puromycin for approximately 1 month. Transient transfections of siRNAs were performed using RNAimax reagent according to the manufacturer’s recommendations (Invitrogen). Anti-Snail (sc-271977), anti-VEGF (sc-7269), anti-p300 (sc-48343), and Anti-*β*-actin (sc-47778HRP) were purchased from Santa Cruz Biotechnology. Anti-Sox2 (ab93689), anti-CD31 (ab32457), anti-Endomucin (ab230018), anti-vWF (ab194405), and anti-H3K27AC (ab4729) were obtained from Abcam. Anti-copGFP (PA5-22688) was obtained from Invitrogen. CD31-FITC (11-0311-82) was obtained from eBioscience. CD44-FITC (555478) and CD24-PE (555428) were purchased from BD biosciences. VEGFR2 inhibitor Vandetanib (T1656) was obtained from Targetmol. VEGF-neutralizing antibody (MAB293-500) were purchased from R&D Systems.

### Cell lineage tracing of breast cancer cells

To perform cell lineage tracing, breast cancer cells were infected with lentivirus carrying fluorescent reporters (copGFP), and then transplanted into nude mice to establish xenografts. Sections of xenografts tumors were immunostained for EC markers and analyzed for colocalization of copGFP and EC markers.

### Clinical samples and immunohistochemistry (IHC)

Fifteen fresh and 69 formalin-fixed breast cancer tissues were collected from the Chinese PLA General Hospital, with the informed consent of patients and with the approval of the Institutional Review Committees of Chinese PLA General Hospital. All cases are female with 24–71 years of age (mean age: 49.4 years). Normal distribution was performed using SPSS13.0. IHC of formalin-fixed paraffin-embedded samples was performed as described previously^35^. Rabbit anti-Sox2 (ab93689), mouse anti-Snail (sc-271977), mouse anti-VEGF (sc-7269), rabbit anti-CD31 (ab32457) were used at dilutions of 1:100 as the primary antibodies for IHC. Snail, Sox2, VEGF, and CD31 score was generated by multiplying the percentage of stained cells (0–100%) by the intensity of the staining (low, 1+; medium, 2+; strong, 3+). The optimal cutoff values of the IHC scores were determined using receiver operating characteristic (ROC) curve analysis. We defined score < 1.5 as low Snail, Sox2, VEGF, and score < 1.0 as low CD31.

### Immunofluorescence

Cells grown on glass coverslips or tissue sections were fixed and permeabilized. Endogenous peroxidase activity was quenched by treatment with 3% H_2_O_2_ for 15 min. Cells or tissue sections were blocked with normal serum for 30 min. The coverslips were then incubated with rabbit anti-CD31 (ab32457), mouse anti-vWF (ab194405), rabbit anti-Endomucin (ab230018), and rabbit anti-copGFP (PA5-22688) at 4 °C overnight. Horse raddish peroxidase (HRP)-conjugated secondary antibodies (DS-0001, PV-6001 and PV-9001 or PV-9003, Zhongshan Biotech) were applied at 37 °C for 1 h, and then immunoreactive cells were visualized with fluorescein amplification reagent (NEL701A001KT, Perkinelmer). Nuclei were counterstained with 4′,6-diamidino-2-phenylindole (DAPI). Confocal images were collected by a Radiance2100 confocal microscope (Bio-Rad).

### Quantitative reverse transcription-PCR (qRT-PCR)

Total RNA was isolated using TRIzol reagent according to the manufacturer’s instructions (Invitrogen). Two micrograms of total RNA was reversely transcribed using SuperScript II Reverse Transcriptase (Invitrogen). qRT-PCR was performed with SYBR-green premix (Takara) on a CFX96 Real-Time PCR detection system. The relative fold change of target mRNAs was normalized to *β*-actin calculated by 2^−∆∆Ct^ method. The primers used for qRT-PCR are listed in Table S7.

### Western blot analysis

Cell pellets were lysed with RIPA lysis buffer containing a protease inhibitor cocktail. Proteins were subsequently separated by 10% or 15% SDS–PAGE and transferred to NC membranes. After blocking for 1 h, membranes were immunoblotted with the indicated antibodies and detected by enhanced chemiluminescence. The bands were detected by ChemiDoc Imaging Systems with Image Lab 2.0 software (Bio-Rad).

### FACS analysis

Cells were collected with 0.25% trypsin-EDTA, and incubated with mouse monoclonal FITC or/and PE-conjugated antibodies against CD31 (11-0311-82, eBioscience), CD24 (555428, BD biosciences), CD44 (555478, BD biosciences). The expression of various specific cell surface markers was analyzed by a flow cytometer (BD Biosciences). FACS data were analyzed with FlowJo software (Treestart).

### ALDEFLUOR assay by FACS

Cells were collected and suspended in ALDEFLUOR assay buffer containing ALDH substrate (StemCell Technologies) and incubated for 30 min at 37 °C. An aliquot of each sample was treated with 50 mmol/l diethylaminobenzaldehyde (DEAB), a specific ALDH inhibitor, as negative control. Cells were analyzed on flow cytometer (BD Biosciences). FACS data were analyzed with FlowJo software (Treestart).

### Tube formation and DiI-AcLDL uptake assay

Ninety-six plates were coated with 50 μl of Matrigel (BD Biosciences) and incubated at 37 °C for 10 min. 2 × 10^4^ cells were plated onto the coated plates and incubated for 6 h at 37 °C. The tube length was measured using Image-Pro Plus. The functional assay for EC was performed by incubation of cells with 10 μg/ml of DiI-AcLDL (Invitrogen) for 4 h followed by observation by fluorescence microscope.

### Mammospheresphere formation assay

Cells were seeded (5000/well) in six-well ultra-low attachment plates (Corning) in DMEM supplemented with 20 ng/ml EGF, 2% B27 supplement, and 20 ng/ml fibroblast growth factor. The number of mammospheres in each well was evaluated after 14 days of culture.

### Dual luciferase reporter assay

Cells were seeded into 24-well plates and cotransfected with luciferase constructs, indicated expression vectors and Renilla luciferase plasmid using Lipofectamine 3000 Reagent. Cells were then lysed and analyzed for luciferase activity with dual-luciferase assay kit (Vigorous) according to the manufacture’s protocol. Firefly luciferase activity was normalized to Renilla luciferase activity as control of transfection efficiency. Relative luciferase activity was detected by luminometer.

### Co-immunoprecipitation

Cells were harvested and lysed in lysis buffer (50 mM Tris at pH 8.0, 500 mM NaCl, 0.5% Nonidet P-40, 1 mM dithiothreitol and protease inhibitors). The protein extracts were then immunoprecipitated with antibody or control serum (Santa Cruz Biotechnology) at 4 °C for 4 h. The precipitated proteins were separated and detected with western blot.

### ChIP assay

ChIP assay was conducted with the Magna ChIP kit according to the manufacturer’s instructions (Millipore). Briefly, nuclear proteins and DNA were cross-linked by incubation in 1% formaldehyde. After sonicating, equal amounts of soluble chromatin were incubated with and without anti-normal rabbit IgG, anti-Snail (sc-271977), anti-p300 (sc-48343), and anti-H3K27AC (ab4729). After antibody incubations, DNA-nuclear proteins cross-linking was reversed and DNA fragments were purified. The purified DNA samples were then amplified by qRT-PCR to determine relative enrichment. DNA fragments obtained without antibody were used as the input controls, and DNA fragments obtained with normal rabbit IgG were applied as negative controls. Primer sequences used are listed in Table S8.

### Enzyme-linked immunosorbent (ELISA)

Breast cancer cells were cultured in serum-free conditions, and the culture supernatants were collected after 72 h. VEGF produced by breast cancer cells was quantified using ELISA kit for human VEGF according to the manufacturer’s recommendations (Invitrogen).

### Animal experiments

Animal studies follow an animal use protocol approved by the Institutional Animal Care and Use Committee of Beijing Institute of Biotechnology. 5 × 10^6^ breast cancer cells isolated from patients or ZR75-1 cells were injected into the abdominal mammary fat pad of 6-week-old female NOD/SCID mice. The mice were randomized into different groups. Six days prior to cell injection, mice were treated with subcutaneous 17*β*-estradiol pellet (0.72 mg). Tumor growth was monitored by caliper measurement every 3 days and the tumor volume was calculated according to the following formula: volume = (longest diameter × shortest diameter^2^)/2.

### Statistical analysis

Trial experiments or similar experiments done previously were used to assess sample size with adequate statistical power. DFS and OS curves were generated by the Kaplan–Meier method and differences between survival curves were determined using the log-rank test. Data were analyzed with two-tailed Student’s *t*-test for two comparisons or one-way ANOVA test with Bonferroni correction for multiple comparisons. All statistical tests were two-sided. Statistical calculations were performed using SPSS 17.0. *P* values of <0.05 were considered statistically significant.

## Supplementary information

Figure S1

Figure S2

Figure S3

Figure S4

Figure S5

Figure S6

Supplementary Figure Legends

Table S1

Table S2

Table S3

Table S4

Table S5

Table S6

Table S7

Table S8

## Data Availability

The authors declare that all data of this study are available within the article and available from the corresponding authors upon request.
