# Supplementary figures and images for: Snail promotes the generation of vascular endothelium by breast cancer cells
Source: Cell Death Dis. 2020 Jun 15;11(6):457. doi: 10.1038/s41419-020-2651-5 (PMC7295784; doi:10.1038/s41419-020-2651-5)

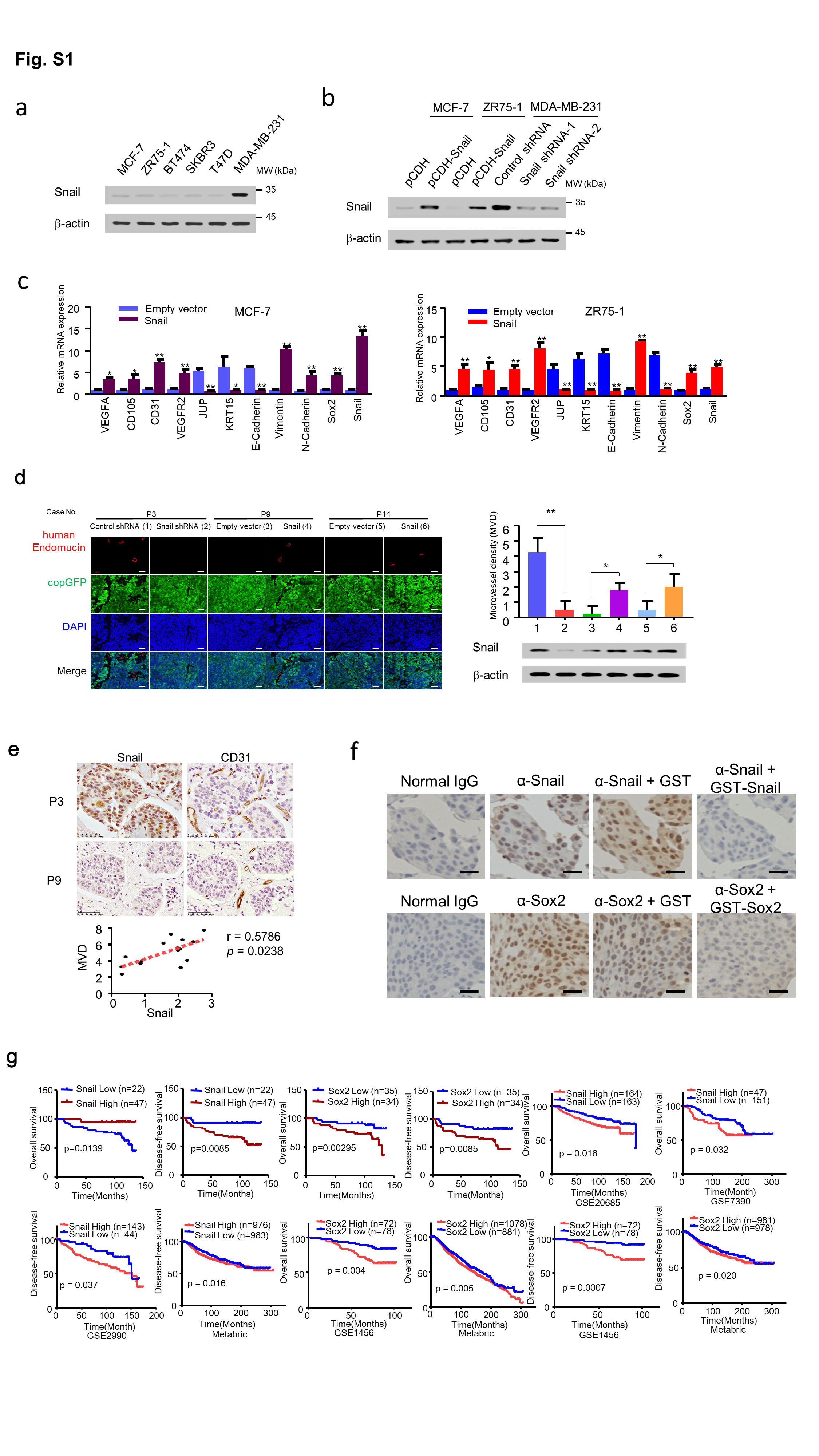

Supplement: Supplementary file 1 — Figure S1 [file 41419_2020_2651_MOESM1_ESM.tif]

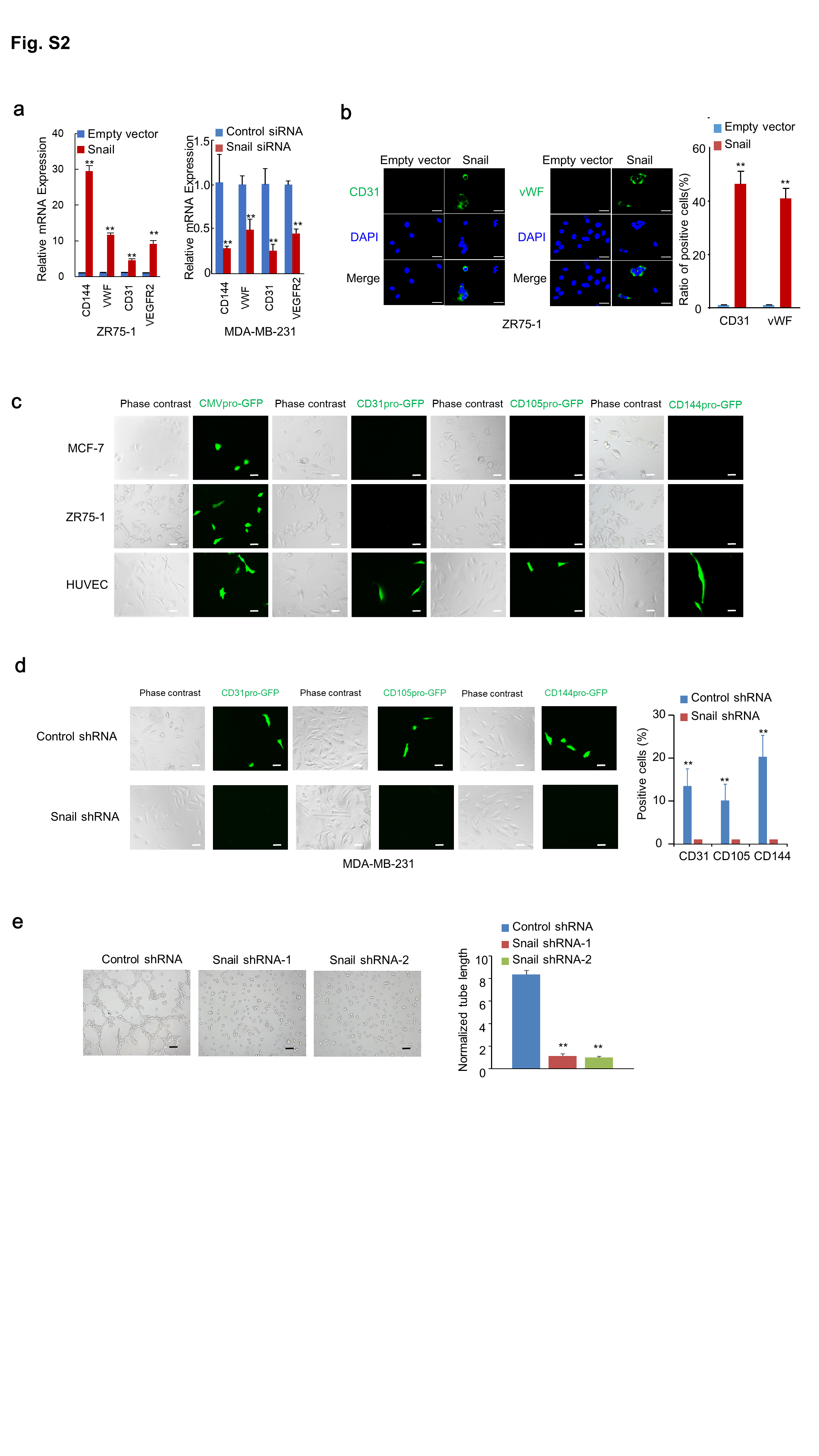

Supplement: Supplementary file 2 — Figure S2 [file 41419_2020_2651_MOESM2_ESM.tif]

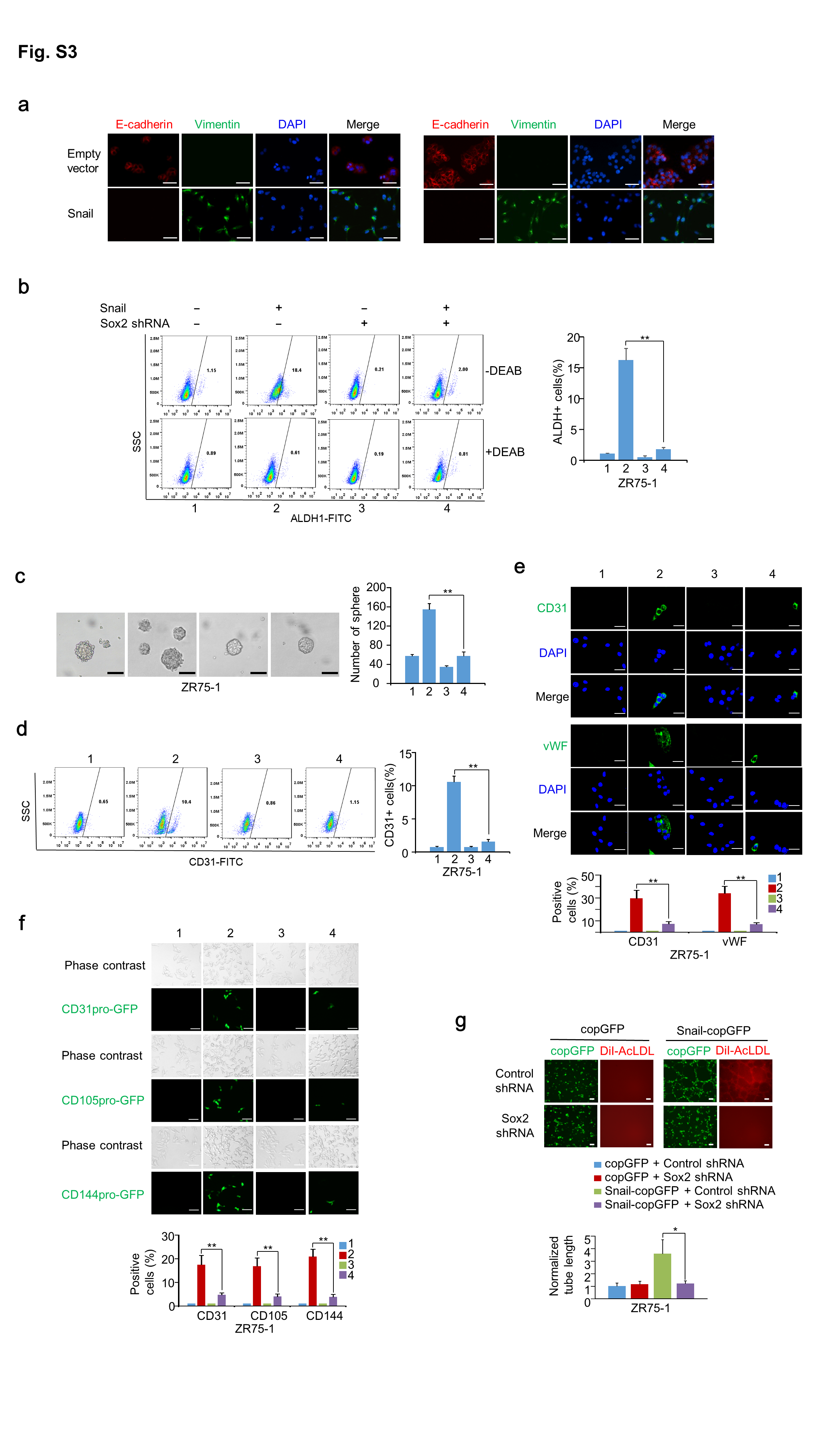

Supplement: Supplementary file 3 — Figure S3 [file 41419_2020_2651_MOESM3_ESM.tif]

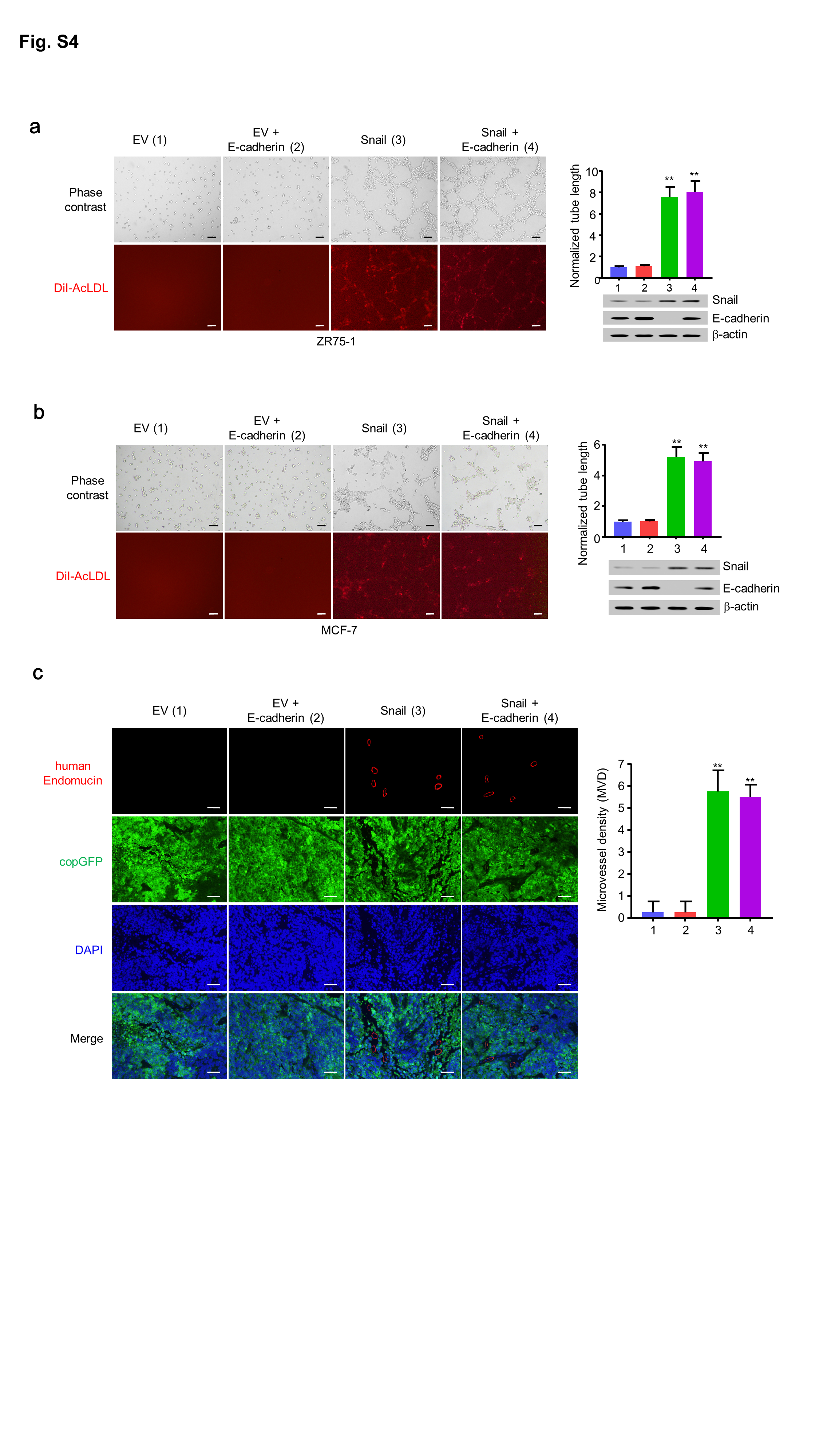

Supplement: Supplementary file 4 — Figure S4 [file 41419_2020_2651_MOESM4_ESM.tif]

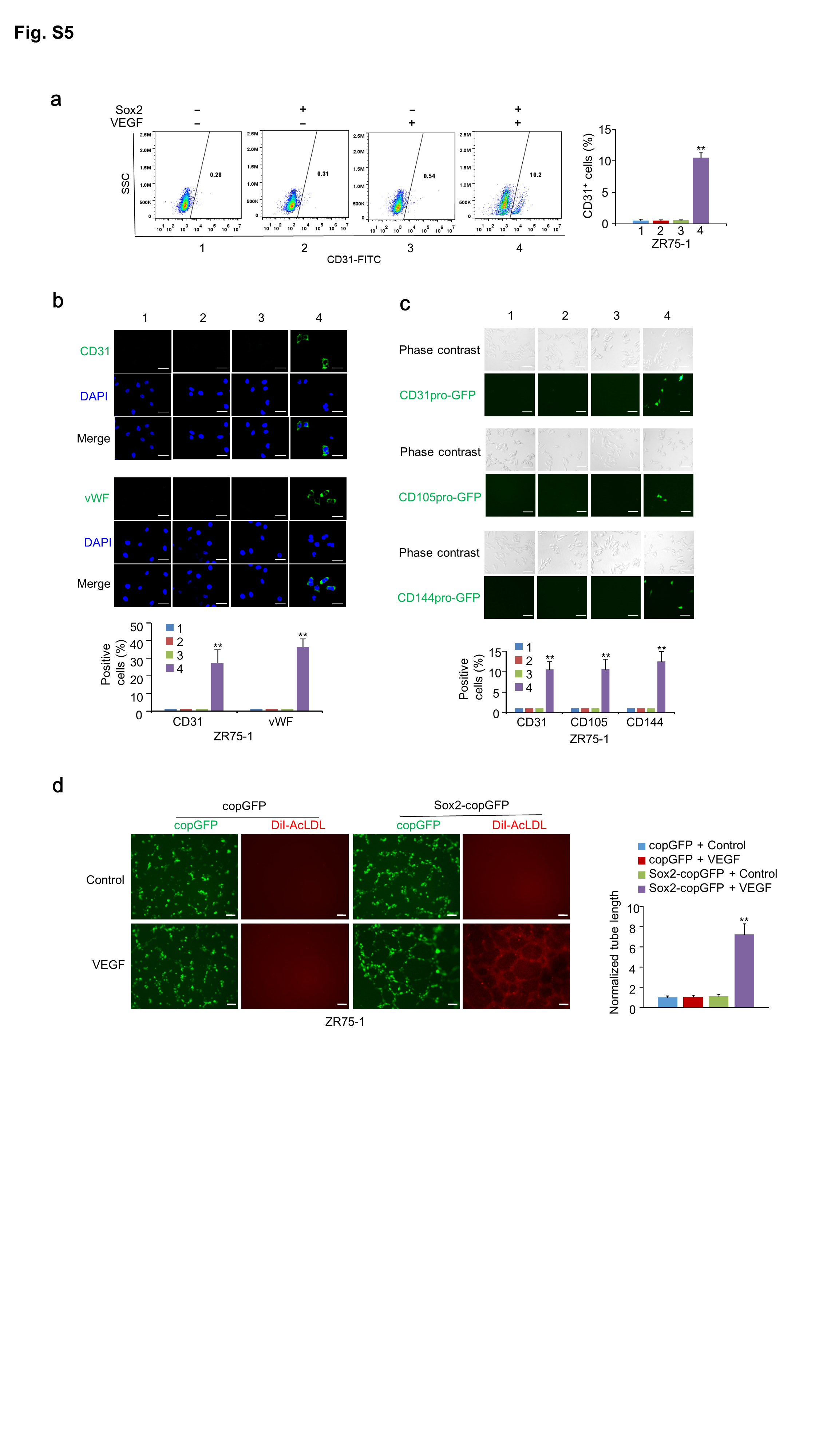

Supplement: Supplementary file 5 — Figure S5 [file 41419_2020_2651_MOESM5_ESM.tif]

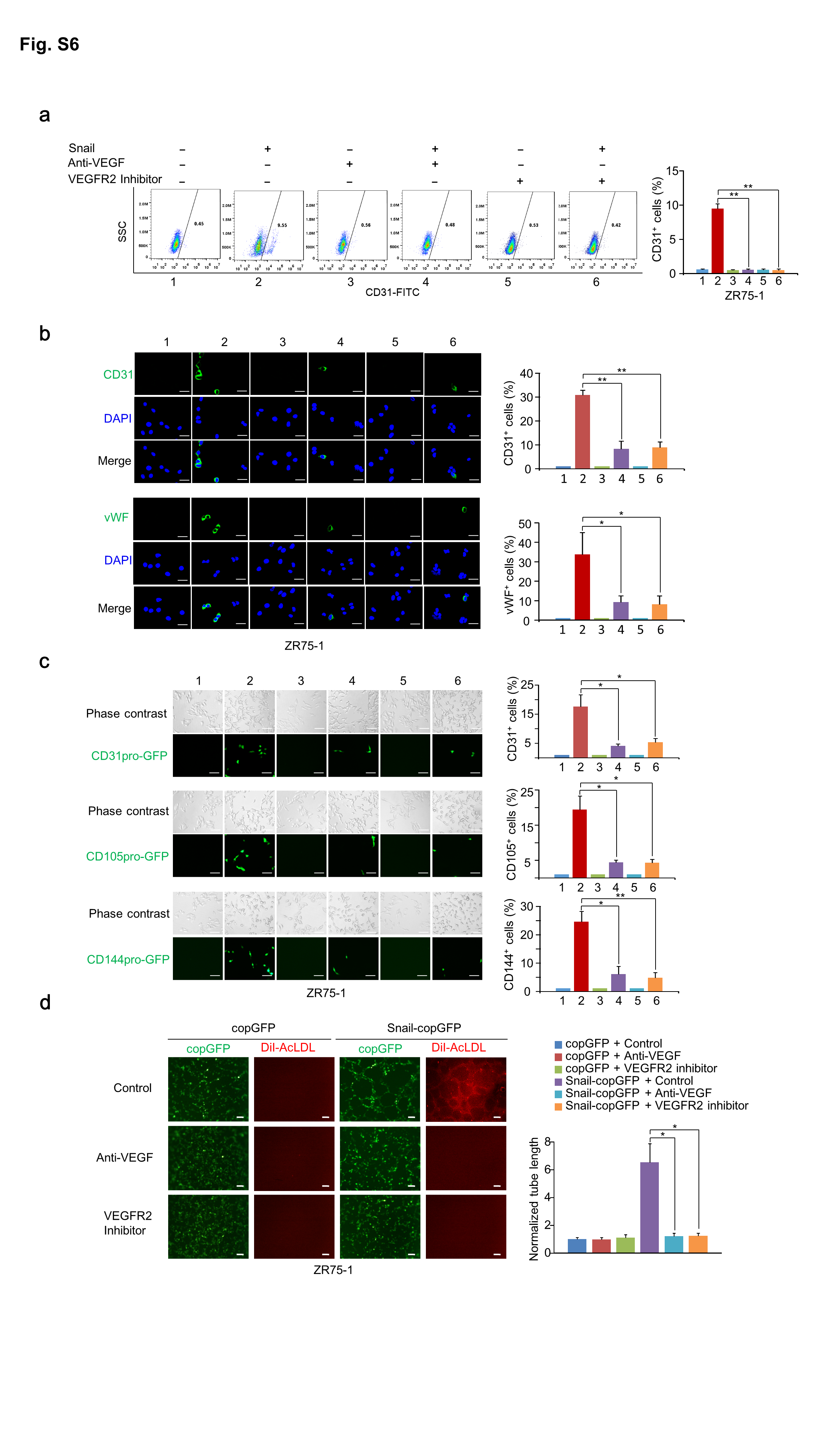

Supplement: Supplementary file 6 — Figure S6 [file 41419_2020_2651_MOESM6_ESM.tif]
