# Supplementary material for: Snail promotes the generation of vascular endothelium by breast cancer cells
Source: Cell Death Dis. 2020 Jun 15;11(6):457. doi: 10.1038/s41419-020-2651-5 (PMC7295784; doi:10.1038/s41419-020-2651-5)
Supplement: Supplementary file 7 — Supplementary Figure Legends [file 41419_2020_2651_MOESM7_ESM.docx]

**Supplementary Figure Legends**

**Fig. S1. Clinical relevance of the Snail and Sox2 in breast cancer. a**. Western blot analysis of Snail expression in breast cancer cell lines as indicated. **b**. Western blot analysis of ectopic Snail in ZR75-1 and MCF-7 cells, and Snail knock-down in MDA-MB-231 cells. **c**. qRT-PCR analysis of EMT- and angiogenesis-related gene expression in MCF-7 and ZR75-1 cells infected with EV or Snail. **d**. Representative IF staining of endomucin and copGFP in xenografts tumors of patients-derived breast cancer cells stably infected with lentivirus carrying Snail shRNA or Snail. Graphs show relative MVD of the indicated groups in the right panel. Data are shown as means ± SD. Western blot analysis of Snail expression in breast cancer cells stably infected with the indicated lentiviruses. β-actin was used as a loading control. *P<0.05, **P<0.01. Scale bar: 50 μm. **e**. Representative immunohistochemical staining of Snail and CD31 in 15 breast cancer samples. The relationship between Snail and MVD was detected by Spearman rank correlation analysis in 15 breast cancer samples. Symbols represent individual samples. **f**. Immunohistochemical staining of breast cancer samples incubated with normal IgG and anti-Snail or anti-Sox2. To validate antibody specificity, the anti-Snail or anti-Sox2 was pre-incubated with recombinant His-Snail or His-Sox2 for 1 h prior to applying to tissues. Scale bar, 50 μm. **g**. The overall survival and disease-free curves related to low and high expression of Snail or Sox2 were analyzed in breast cancer patients from the GEO database and samples we collected.

**Fig. S2. Snail induces endothelium generation of breast cancer cells**. **a**. qRT-PCR assay of the relative mRNA expression of endothelium markers in ZR75-1 cells stably infected with lentivirus carrying EV or Snail, and in MDA-MB-231 cells transfected with negative control or Snail siRNA. **b**. Representative confocal images of ZR75-1 cells stably infected with lentivirus carrying EV or Snail immunostained with endothelium markers CD31 and vWF. The nuclei were stained with DAPI (blue). Scale bar, 20 μm. Graphs show the percentage of endothelium marker-positive cells. Results shown are mean ± SD of three independent experiments. **c**. Cell type-specific expression of the CD31-, CD105-, or CD144-promoter-driven GFP constructs in vitro functional test. Endothelial cells (HUVEC) and epithelial cells (MCF-7 and ZR75-1) were infected with the indicated lentiviral vectors carrying the indicated promoters or GFP. Scale bar, 5 μm. **d**. Endothelium lineage tracing of MDA-MB-231 cells infected with negative control or Snail shRNAs with CD31pro-GFP, CD105pro-GFP, or CD144pro-GFP. Scale bar, 5 μm. Statistical analysis of GFP-positive rates was is shown in the right panel. Results shown are mean ± SD of three independent experiments. **e**. Tube-forming assays with MDA-MB-231 cells infected with negative control or Snail shRNAs. Statistical analysis of tube length was is shown in the right panel. Results shown are mean ± SD of three independent experiments. Scale bar, 100 μm.

**Fig. S3**. **Snail induces stemness properties and endothelium generation of breast cancer cells through Sox2**. **a.** IF of ZR75-1 and MCF-7 cells stably infected with lentivirus carrying EV or Snail using E-cadherin and Vimentin antibodies. The nuclei were stained with DAPI (blue). Scale bar, 20 μm. **b**. Representative FACS with the ALDH1-FITC with or without DEAB in ZR75-1 cells infected with lentivirus carrying vectors as (a). Statistical analysis of ALDHL1-positive rates was is shown in the right panel. Results shown are mean ± SD of three independent experiments. **c**. Mammosphere assay of ZR75-1 cells infected with lentivirus carrying vectors plasmids as (a). Graphs show the relative number of mammospheres. Results shown are mean ± SD of three independent experiments. Scale bar, 20 μm. **d**. FACS analysis of ZR75-1 cells infected with lentivirus carrying vectors as (a) with CD31-FITC. Statistical analysis of CD31+ rates is shown in the right panel. Results shown are mean ± SD of three independent experiments. **e**. Representative confocal images of ZR75-1 cells infected with vectors plasmids as (a) labelled with endothelium markers CD31 and vWF. The nuclei were stained with DAPI (blue). Scale bar, 20 μm. Graphs show the percentage of endothelium marker- positive cells. Results shown are mean ± SD of three independent experiments. **f**. Lineage tracing of ZR75-1 cells stably expressing EV or Snail with CD31pro-GFP, CD105pro-GFP, or CD144pro-GFP. Scale bar, 10 μm. Graphs show the percentage of GFP-positive cells. Results shown are mean ± SD of three independent experiments. **g**. Tube-forming assays and DiI-AcLDL uptake assays with Snail-copGFP or copGFP- overexpressing ZR75-1 cells. Statistical analysis of tube length was is shown in the right panel. Results shown are mean ± SD of three independent experiments. Scale bar, 100 μm. Red: DiI-AcLDL staining.

**Fig. S4**. **Snail-mediated angiogenesis is independent of E-cadherin**. **a-b.** Tube-forming and DiI-AcLDL uptake assays with ZR75-1 cells (a) and MCF-7 cells (b) infected with lentivirus carrying E-cadherin, Snail or E-cadherin plus Snail. Statistical analysis of tube length is shown in the right panel. Results shown are mean ± SD of three independent experiments. Scale bar, 100 μm. Red: DiI-AcLDL staining. Western blot analysis of Snail and E-cadherin expression in ZR75-1 and MCF-7 cells stably infected with the indicated lentiviruses. β-actin was used as a loading control. **c**. Representative IF staining of xenograft tumors of ZR75-1 cells infected with lentivirus carrying E-cadherin, Snail or E-cadherin plus Snail with anti-human endothelium marker endomucin (red) and copGFP (green). Scale bar: 50 μm. Graphs show relative MVD of the indicated groups in the right panel. Data are shown as means ± SD. *P<0.05, **P<0.01.

**Fig. S5. Sox2 alone fails to induce endothelium generation in breast cancer cells. a.** FACS analysis with CD31-FITC antibodies in EV- or Sox2-stably expressing ZR75-1 cells cultured with or without VEGF. Statistical analysis of CD31+ rates is shown in the right panel. Results shown are mean ± SD of three independent experiments. **b**. Representative confocal images of endothelium marker in ZR75-1 cells treated as (a). The nuclei were stained with DAPI (blue). Scale bar, 20 μm. The graphs show the percentage of endothelium marker-positive cells. Results shown are mean ± SD of three independent experiments. **c**. Lineage tracing of ZR75-1 cells treated as (a) using CD31pro-GFP, CD105pro-GFP, or CD144pro-GFP cultured with or without VEGF. Scale bar, 10 μm. Graphs show the percentage of GFP-positive cells. Results shown are mean ± SD of three independent experiments. **d**. Tube-forming assays and DiI-AcLDL uptake assays with copGFP- or SOX2-copGFP-overexpressing ZR75-1 cells cultured with or without VEGF. Statistical analysis of tube length is shown in the right panel. Results shown are mean ± SD of three independent experiments. Scale bar, 100 μm. Red: DiI-AcLDL staining.

**Fig. S6. Endothelium generation of breast cancer cells induced by Snail depends on VEGF signaling. a.** Representative FACS using CD31-FITC of ZR75-1 cells stably expressing EV or Snail treated with or without VEGF neutralized antibody or VEGFR2 inhibitor. Graphs show the percentage of CD31-positive cells. Results shown are mean ± SD of three independent experiments. **b**. IF analysis of ZR75-1 cells treated as (a) with CD31 and vWF antibodies. Graphs show the percentage of CD31- or VWF-positive cells. Results shown are mean ± SD of three independent experiments. **c**. Lineage tracing of ZR75-1 cells treated as (a) with CD31pro-GFP, CD105pro-GFP, or CD144pro-GFP. Scale bar, 10 μm. Graphs show the percentage of GFP-positive cells. Results shown are mean ± SD of three independent experiments. **d**. Tube-forming assays and DiI-AcLDL uptake assays with copGFP- or Snail-copGFP-expressing ZR75-1 cells treated with or without VEGF neutralized antibody or VEGFR2 inhibitor. Statistical analysis of tube length is shown in the right panel. Results shown are mean ± SD of three independent experiments. Scale bar, 100 μm. Red: DiI-AcLDL staining.
