# Supplementary material for: Snail promotes the generation of vascular endothelium by breast cancer cells
Source: Cell Death Dis. 2020 Jun 15;11(6):457. doi: 10.1038/s41419-020-2651-5 (PMC7295784; doi:10.1038/s41419-020-2651-5)
Supplement: Supplementary file 8 — Table S1 [file 41419_2020_2651_MOESM8_ESM.docx]

**Table S1: Characteristics of specimens used in the study**

| Case  No. | Age | Stage | Tumors  tatus | Nodal  status | ER | PR | HER2 |
| --- | --- | --- | --- | --- | --- | --- | --- |
|  |  |  |  |  | (1:positive;0:negative) | | |
| 1 | 42.7 | Ⅲa | T2 | N2 | 1 | 1 | 1 |
| 2 | 52.4 | Ⅰ | T1 | N0 | 1 | 1 | 1 |
| 3 | 24.5 | Ⅲc | T2 | N3 | 0 | 0 | 0 |
| 4 | 51.3 | Ⅱb | T3 | N0 | 0 | 0 | 1 |
| 5 | 36.8 | Ⅲa | T2 | N2 | 0 | 0 | 0 |
| 6 | 38.3 | Ⅱb | T2 | N1 | 1 | 1 | 0 |
| 7 | 44.9 | Ⅲa | T3 | N1 | 1 | 1 | 0 |
| 8 | 44.1 | Ⅱa | T2 | N0 | 1 | 1 | 0 |
| 9 | 50.3 | Ⅰ | T1 | N0 | 1 | 1 | 0 |
| 10 | 43.5 | Ⅱa | T2 | N0 | 0 | 0 | 0 |
| 11 | 45.4 | Ⅰ | T1 | N0 | 1 | 1 | 0 |
| 12 | 36.4 | Ⅱa | T1 | N1 | 1 | 0 | 0 |
| 13 | 42.4 | Ⅲa | T3 | N2 | 0 | 0 | 0 |
| 14 | 67.2 | Ⅲb | T4 | N2 | 1 | 1 | 0 |
| 15 | 54.7 | Ⅱb | T2 | N1 | 1 | 1 | 1 |
