# Supplementary material for: Snail promotes the generation of vascular endothelium by breast cancer cells
Source: Cell Death Dis. 2020 Jun 15;11(6):457. doi: 10.1038/s41419-020-2651-5 (PMC7295784; doi:10.1038/s41419-020-2651-5)
Supplement: Supplementary file 9 — Table S2 [file 41419_2020_2651_MOESM9_ESM.docx]

**Table S2.** **Correlation between Snail and Sox2 or angiogenesis-related genes expression in breast cancer patients**

Correlation of Snail and Sox2

| **Dataset** | **Correlation coefficient** | **Q value** |
| --- | --- | --- |
| GSE1456 | 0.376151 | 1.13E-05 |
| GSE2034 | 0.359841 | 3.47E-09 |
| GSE5847 | 0.603901 | 2.69E-04 |
| GSE11121 | 0.380502 | 2.95E-07 |
| GSE12093 | 0.335118 | 9.72E-04 |
| GSE20194 | 0.519771 | 1.08E-19 |
| GSE20271 | 0.541071 | 4.92E-13 |
| GSE25055 | 0.513213 | 2.17E-22 |

Correlation of Snail and Endothelial-related genes

| **Gene** | **Dataset** | **Correlation coefficient** | **Q value** |
| --- | --- | --- | --- |
| VEGFA | GSE20685 | 0.331635 | 2.47E-08 |
| VEGFC | TCGA RNAseq | 0.315003 | 1.76E-25 |
| CD31 | GSE20194 | 0.321166 | 1.66E-07 |
|  | GSE25055 | 0.330566 | 3.91E-09 |
|  | TCGA RNAseq | 0.318133 | 5.38E-26 |
| CD105 | GSE19615 | 0.335705 | 5.38E-03 |
|  | TCGA RNAseq | 0.4135428 | 3.84E-45 |
| Endomucin | GSE20194 | 0.303528 | 8.58E-07 |
