# Supplementary material for: Snail promotes the generation of vascular endothelium by breast cancer cells
Source: Cell Death Dis. 2020 Jun 15;11(6):457. doi: 10.1038/s41419-020-2651-5 (PMC7295784; doi:10.1038/s41419-020-2651-5)
Supplement: Supplementary file 10 — Table S3 [file 41419_2020_2651_MOESM10_ESM.docx]

**Table S3: Correlation of Snail1 and Sox2 status with clinical factors***

| Clinical characteristics | Total cases | Snail  low | Snail  high | *P* value | SOX2  low | SOX2  high | *P* value |
| --- | --- | --- | --- | --- | --- | --- | --- |
| Age  ≤ 50 years  > 50 years | 40  29 | 13  14 | 27  15 | 0.185 | 19  17 | 21  12 | 0.361 |
| Tumor size  ≤ 20 mm  > 20 mm | 35  34 | 18  9 | 17  25 | 0.034 | 23  13 | 12  21 | 0.022 |
| Nodal status  Negative  Positive  Grade  I  II  III | 38  31  15  31  23 | 19  8  12  8  7 | 19  23  3  23  16 | 0.041  0.001 | 25  11  12  16  8 | 13  20  3  15  15 | 0.012  0.024 |
| ERα  Negative  Positive | 23  46 | 13  14 | 10  32 | 0.036 | 14  22 | 9  24 | 0.307 |
| PR  Negative  Positive | 29  40 | 8  19 | 21  21 | 0.094 | 11  25 | 18  15 | 0.044 |
| HER2  Negative  Positive | 50  19 | 22  5 | 28  14 | 0.179 | 28  8 | 22  11 | 0.302 |

**P* values were assessed by Pearson chi-square test.
