# Supplementary material for: Snail promotes the generation of vascular endothelium by breast cancer cells
Source: Cell Death Dis. 2020 Jun 15;11(6):457. doi: 10.1038/s41419-020-2651-5 (PMC7295784; doi:10.1038/s41419-020-2651-5)
Supplement: Supplementary file 11 — Table S4 [file 41419_2020_2651_MOESM11_ESM.docx]

**Table S4: Cox survival analysis of disease-free survival and overall survival in patients with breast cancer**

|  | Factor | *P* value | HR | 95% CI | |
| --- | --- | --- | --- | --- | --- |
| Disease-free survival | Tumor size: ≤20 vs > 20 mm | 0.0001 | 11.783 | 3.395-40.894 | |
|  | Lymph node: negative vs positive  Grade: I vs II vs III | 0.006  0.009 | 3.773  2.408 | 1.472-9.672  1.251-4.633 |  |
|  | ER: negative vs positive | 0.030 | 0.392 | 0.168-0.915 |  |
|  | PR: negative vs positive | 0.014 | 0.336 | 0.140-0.805 |  |
|  | HER2: negative vs positive | 0.056 | 2.290 | 0.978-5.366 |  |
|  | Snail: negative vs positive | 0.025 | 3.451 | 1.166-10.210 |  |
|  | SOX2: negative vs positive | 0.071 | 2.225 | 0.933-5.308 |  |
| Overall survival | Tumor size: ≤20 vs > 20 mm | 0.001 | 13.448 | 3.037-59.550 | |
|  | Lymph node: negative vs positive  Grade: I vs II vs III | 0.007  0.003 | 4.666  3.253 | 1.533-14.199  1.479-7.157 |  |
|  | ER: negative vs positive | 0.030 | 0.343 | 0.131-0.901 |  |
|  | PR: negative vs positive | 0.088 | 0.437 | 0.169-1.131 |  |
|  | HER2: negative vs positive | 0.029 | 2.820 | 1.109-7.172 |  |
|  | Snail: negative vs positive | 0.046 | 3.542 | 1.024-12.251 |  |
|  | SOX2: negative vs positive | 0.167 | 1.953 | 0.755-5.050 |  |
