# Supplementary material for: Snail promotes the generation of vascular endothelium by breast cancer cells
Source: Cell Death Dis. 2020 Jun 15;11(6):457. doi: 10.1038/s41419-020-2651-5 (PMC7295784; doi:10.1038/s41419-020-2651-5)
Supplement: Supplementary file 12 — Table S5 [file 41419_2020_2651_MOESM12_ESM.docx]

**Table S5. Primers sequence for promoters and their mutations**

| **Sox2 promoter** | **Forward** | | **Reverse** |
| --- | --- | --- | --- |
| -798~+50 | GGGGTACCGCTAAGAGGAAGAGCTGCAG | | CCCAAGCTTGCCTTGACAACTCCTGATACT |
| site A mutant (-569/-564)  site B mutant (-6/-1) | GAGAGTGTTGGTGTTCATAAGGTA  AGCCCCCGTTGTGCAGATGGTTGTCTA | | TACCTTATGAACACCAACACTCTC  TAGACAACCATCTGCACAACGGGGGCT |
| **VEGF promoter** | | **Forward** | **Reverse** |
| -1126~+72 | | CCGCTCGAGAATCATCACGCAGGCCCTGGC | CCCAAGCTTTCCCGACAGAGCGCTGGTGC |
| site A mutant (-1017/-1012) | | GGGGTACCGCTAAGAGGAAGAGCTGCAG | AAAGGGGGAGGTGATCAGGCAGACAGA |
| site B mutant (-654/-649) | | AATAGGGGGTCTGAAGACAAACTCCC | GGGAGTTTGTCTTCAGACCCCCTATT |
| **CD144 promoter** | | **Forward** | **Reverse** |
| -862~+50 | | CCATCGATGTCACTTGACTCCACCCAAT | GCTCTAGACTTCCCAGGAGGAACAGATC |
