# Supplementary material for: Snail promotes the generation of vascular endothelium by breast cancer cells
Source: Cell Death Dis. 2020 Jun 15;11(6):457. doi: 10.1038/s41419-020-2651-5 (PMC7295784; doi:10.1038/s41419-020-2651-5)
Supplement: Supplementary file 13 — Table S6 [file 41419_2020_2651_MOESM13_ESM.docx]

**Table S6. The cDNA target sequences of shRNA and siRNA**

| Gene | Target sequence (5’-3’) |
| --- | --- |
| Sox2 | GGAATGGACCTTGTATAGATC |
| Snail | CACGAGGTGTGACTAACTATT  GCTGCAGGACTCTAATCCAGA |
| p300 | AGATGAGAGTTTAGGCCGC |
| VEGF | GGCCAGCACATAGGAGAGA |
