# Supplementary material for: Snail promotes the generation of vascular endothelium by breast cancer cells
Source: Cell Death Dis. 2020 Jun 15;11(6):457. doi: 10.1038/s41419-020-2651-5 (PMC7295784; doi:10.1038/s41419-020-2651-5)
Supplement: Supplementary file 14 — Table S7 [file 41419_2020_2651_MOESM14_ESM.docx]

**Table S7. Primers sequence for qRT-PCR**

| **Gene** | **Forward primer (5’-3’)** | **Reverse primer (5’-3’)** |
| --- | --- | --- |
| VEGFA | AGAGCAAGACAAGAAAATCC | TACAAACAAATGCTTTCTCC |
| CD105 | GAACACAAAGAACATAGTTGG | AAGCCAACTGCACTTGCATTG |
| VEGFR2  CD31  CD144  vWF | TATACAGCAAGCGACTGAATG  GCAACACAGTCCAGATAGTCGT  AAGCCTCTGATTGGCACAGT  CCACAAGGTCATTTCTCCAGCCAC | GTAGTGCTGTGCTGCTGCCAC  GACCTCAAACTGGGCATCAT  CTGGCCCTTGTCACTGGT  GGTCCGACAGAGGTGAGCATAAG |
| JUP | TCTCCAACCTGACATGCAACA | CATAGTTGAGACGCACAGAGTTC |
| KRT15 | GACGGAGATCACAGACCTGAG | CTCCAGCCGTGTCTTTATGTC |
| E-Cadherin | GAAAGCGGCTGATACTGACC | CGTACATGTCAGCCGCTTC |
| Vimentin | TCTACGAGGAGGAGATGCGG | GGTCAAGACGTGCCAGAGAC |
| N-Cadherin | TGTTTGACTATGAAGGCAGTGG | TCAGTCATCACCTCCACCAT |
| Sox2 | CCCAGCAGACTTCACATGT | CCTCCCATTTCCCTCGTTTT |
| Snail | ACTGCAACAAGGAATACCTCAG | GCACTGGTACTTCTTGACATCTG |
