# Supplementary material for: Snail promotes the generation of vascular endothelium by breast cancer cells
Source: Cell Death Dis. 2020 Jun 15;11(6):457. doi: 10.1038/s41419-020-2651-5 (PMC7295784; doi:10.1038/s41419-020-2651-5)
Supplement: Supplementary file 15 — Table S8 [file 41419_2020_2651_MOESM15_ESM.docx]

**Table S8. Primers sequence for ChIP**

| **Sox2 promoter** | **Forward** | **Reverse** |
| --- | --- | --- |
| Site A | TTATGGTCCGAGCAGGATTT | GCTCTTCCGCTCTCCTCTCT |
| Site B | CGCTGATTGGTCGCTAGAA | GCCTTGACAACTCCTGATACTTT |
| **VEGF promoter** | **Forward** | **Reverse** |
| Site A | CTCAGTTCCCTGGCAACATC | CACCAAGTTTGTGGAGCTGA |
| Site B | GAAGCAACTCCAGTCCCAAA | CACACACGTCCTCACTCTCG |
